# User appraisal of a booklet for advance care planning in multiple sclerosis: a multicenter, qualitative Italian study

**DOI:** 10.1007/s10072-023-07087-y

**Published:** 2023-10-10

**Authors:** Andrea Giordano, Ludovica De Panfilis, Simone Veronese, Michela Bruzzone, Marta Cascioli, Mariangela Farinotti, Ambra Mara Giovannetti, Maria Grazia Grasso, Paola Kruger, Alessandra Lugaresi, Leigh Manson, Marta Perin, Eugenio Pucci, Claudio Solaro, Luca Ghirotto, Alessandra Solari

**Affiliations:** 1 Unit of Neuroepidemiology, Fondazione IRCCS Istituto Neurologico Carlo Besta, Via Celoria 11, 20133 Milan, Italy; 2 Bioethics Unit - Scientific Directorate, Azienda USL-IRCCS di Reggio Emilia, 42100 Reggio Emilia, Italy; 3 Fondazione FARO ETS, 10125 Turin, Italy; 4 Fondazione Italiana Sclerosi Multipla, 16126 Genoa, Italy; 5 Hospice ‘La Torre Sul Colle, Azienda USL Umbria 2, 06049 Spoleto, Italy; 6 Multiple Sclerosis Unit, IRCCS Santa Lucia Foundation, 00179 Rome, Italy; 7 The European Patients’ Academy (EUPATI), 00165 Rome, Italy; 8 UOSI Riabilitazione Sclerosi Multipla, IRCCS Istituto delle Scienze Neurologiche di Bologna, 40121 Bologna, Italy; 9 Dipartimento di Scienze Biomediche e Neuromotorie, Università di Bologna, 40121 Bologna, Italy; 10 Health Quality & Safety Commission New Zealand, 7045 Nelson, New Zealand; 11 Doctoral Program in Clinical and Experimental Medicine, University of Modena and Reggio Emilia, 41100 Modena, Italy; 12 UOC Neurologia AV4, ASUR Marche, 63900 Fermo, Italy; 13 Department of Rehabilitation, CRRF “Mons. L. Novarese”, Loc. Trompone, 13040 Moncrivello, Italy; 14 Qualitative Research Unit, Azienda USL-IRCCS di Reggio Emilia, 42100 Reggio Emilia, Italy

**Keywords:** Multiple sclerosis, Advance care planning, Goals of care, Palliative care, Qualitative research

## Abstract

**Objectives:**

Implementation of advance care planning (ACP) in people with progressive multiple sclerosis (PwPMS) is limited. We aimed to involve users (PwPMS, significant others, and healthcare professionals involved in PwPMS care) in the evaluation and refinement of a booklet to be used during the ACP conversations.

**Methods:**

This qualitative study consisted of cognitive interviews with PwPMS and significant others and a focus group with healthcare professionals from three Italian centers. We analyzed the interviews using the framework method and the focus group using thematic analysis.

**Results:**

We interviewed 10 PwPMS (3 women; median age 54 years; median Expanded Disability Status Scale score 6.0) and three significant others (2 women; 2 spouses and one daughter). The analysis yielded three themes: booklet comprehensibility and clarity, content acceptability and emotional impact, and suggestions for improvement. Twelve healthcare professionals (7 neurologists, 3 psychologists, one nurse, and one physiotherapist) participated in the focus group, whose analysis identified two themes: booklet’s content importance and clarity and challenges to ACP implementation. Based on analysis results, we revised the booklet (text, layout, and pictures) and held a second-round interviews with two PwPMS and one significant other. The interviewees agreed on the revisions but reaffirmed their difficulty in dealing with the topic and the need for a physician when using the booklet.

**Conclusions:**

Appraisal of the booklet was instrumental in improving its acceptability and understandability before using it in the ConCure-SM feasibility trial. Furthermore, our data reveal a lack of familiarity with ACP practice in the Italian context.

**Supplementary Information:**

The online version contains supplementary material available at 10.1007/s10072-023-07087-y.

## Introduction

Multiple sclerosis (MS) is the most common cause of progressive neurological disability in young adults [1, 2]. Approximately 15% of people with MS have a primary progressive course at diagnosis, and an additional 35% develop secondary progressive disease after 15 years [3]. A mean reduction in life expectancy by 7–14 years has been reported, with improved figures over the last two decades [4–6]. Nevertheless, people with progressive MS (PwPMS) may live for many years experiencing a wide range of symptoms, impairments (including cognitive impairment, which affects 40–70% of sufferers), and comorbidities [6–11].

In this context, advance care planning (ACP) is necessary to ensure that PwPMS future care, especially at the end of life (EOL), is consistent with evidence-based practice and a person-value-centered approach [12–15]. ACP “supports adults at any age or health stage in understanding and sharing their personal values, life goals and preferences regarding treatments and future medical care” [16].

Increasing patient and public awareness of the role of ACP for improving the quality of care at the EOL is also crucial. The National Health System has a key role in providing a context for such engagement and in promoting ACP effective use [16]. Initiatives to foster the implementation of ACP have increased over the last decade and include educational programs in the healthcare setting, public awareness campaigns, and national laws [17].

Evidence from non-neurological progressive and life-threatening illnesses shows that ACP helps manage the care path, decreases unwanted life-sustaining treatments, and increases the use of hospice and palliative care [18, 19], as well as alignment with patients’ EOL preferences [19].

Nevertheless, ACP implementation has shown several challenges: it can produce an emotional burden due to thinking about death and the necessity of making plans. From healthcare professionals’ point of view, the length of time to devote to ACP discussion, the difficulty in prognostic prediction, and the disagreement about care goals among multidisciplinary team members have been reported as barriers [20, 21]. A general lack of knowledge on ACP due to inadequate training of healthcare professionals on EOL care, advance directives, and ACP has also been reported [22].

The recently published European Academy of Neurology Guideline on Palliative Care in People with Severe Progressive MS includes the following good practice statement: “It is suggested that early discussion on the future with ACP is offered to patients with severe MS” [23, 24]. This statement was based on consensus between task force members, as no evidence was found on the effectiveness of ACP in PwPMS [23–25]. PwPMS want to talk about their future with healthcare professionals; however, they do not have the opportunity to start these conversations [26–28]. The reasons are complex, and include the unpredictable trajectory of MS and the healthcare professional reluctance to discuss disease progression and care pathways at the EOL. In a qualitative study by Koffman et al., MS patients reported negative experiences of EOL-related discussions with healthcare professionals [29].

ConCure-SM is a multi-phased project aimed to construct and test the efficacy of an MS-specific ACP intervention, consisting of a training program on ACP for healthcare professionals caring for PwPMS and a booklet to be used during the ACP conversation [30]. The training program includes a residential module and an on-site module. The residential module (one-and-half day duration, continuing medical education accredited) includes a theoretical session on the clinical, ethical, and statutory principles of shared decision-making and ACP; two empirical sessions on conducting ACP conversations in various clinical scenarios using the ConCure-SM booklet through guided role play exercises; and two self-evaluation sessions. The on-site module supports healthcare professionals at the centers on issues concerning the conduction of ACP conversations during the feasibility trial. A web-based trial platform contains the trial case record forms and the self-reported outcome measures [30].

**Fig. 1:**
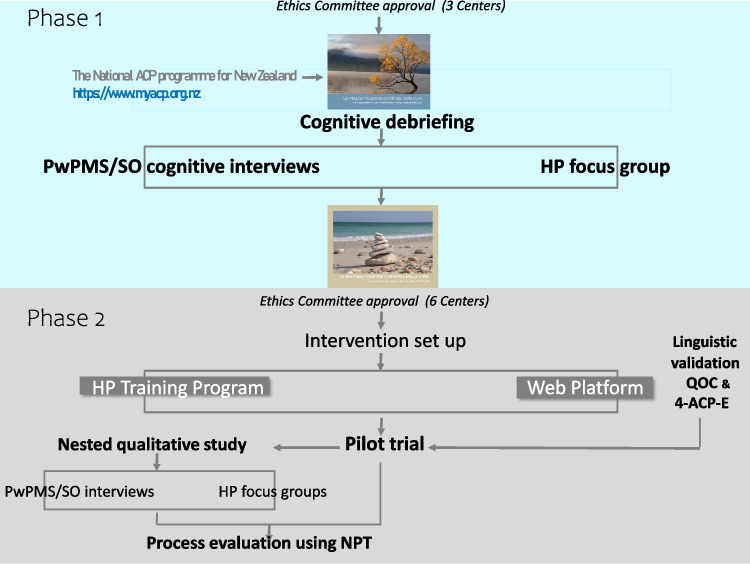
Flow chart of the ConCure-SM project. The two pictures embedded are cover miniatures of the provisional and final booklet. ACP, advance care planning; 4-ACP-E, 4-item ACP Engagement (outcome measure); HP, health professional; MS, multiple sclerosis; NPT, normalization process theory; PwPMS, people with progressive MS; QOC, quality of communication (outcome measure); SO, significant other

In ConCure-SM project phase 1, we co-produced the ACP booklet. In phase 2, we set up the intervention (which includes the booklet), which is currently being tested for efficacy in the feasibility trial (Fig. 1).

The present study refers to project phase 1. Specifically, our objectives were to assess the acceptability and understandability of the ACP booklet for users (PwPMS, significant others, and healthcare professionals) and to refine the booklet accordingly.

## Methods

We conducted a multicenter, qualitative study applying the cognitive interview technique with PwPMS and significant others and a focus group meeting with healthcare professionals. We followed the consolidated criteria for reporting qualitative research (COREQ) (online supplemental file 1).

For PwPMS and significant others, one-to-one cognitive interviews were considered the most appropriate to limit the interview burden and to make it easier for participants to express their feelings. Conceived by cognitive psychologists [31], the cognitive interview technique is traditionally used to evaluate comprehension issues in questionnaire design and to improve self-reported instruments [32]. This technique has also been used to test the understanding of patient information leaflets [33–35].

For the healthcare professionals, we chose the focus group to promote interaction and exchange of ideas.

### Booklet development

In 2020, an interdisciplinary panel, including an expert patient and a representative of the Italian MS Society, translated and adapted the ACP booklet of the Health Quality & Safety Commission’s New Zealand National ACP Program (online supplemental file 2). The resulting booklet in its provisional version (online supplemental file 3) comprised an introduction, a “guidance,” and the ACP document (the even pages) to be completed electronically or manually by PwPMS together with the referring physician. The introduction explained ACP concepts and advance directives according to the Italian Law 219/2017 and described why ACP is important in MS. Ten fillable sections followed: “My advance care plan,” “What matters to me,” “What worries me,” “Why I’m making an advance care plan,” “How I make decisions,” “If I were no longer able to make decisions: my trustee,” “Thinking about my EOL,” “My treatment and care choices,” “Signatures,” and “Abbreviations.”

### Research settings and sampling

The study involved three Italian MS research centers: the Fondazione IRCCS Istituto Neurologico Carlo Besta, Milan; the Azienda USL-IRCCS of Reggio Emilia; and the IRCCS Santa Lucia Foundation, Rome.

PwPMS and significant others aged 18 years or older and fluent in Italian were eligible. PwPMS had to be diagnosed for 1 or more years, able to communicate, and without severe cognitive impairment (clinician’s judgement); they were purposely selected as assorted in age, gender, disability status, age at MS diagnosis, and age at progression. Eligible PwPMS and significant others were invited to participate by neurologists at each participating center. Participants who provided informed consent were contacted by phone or e-mail by an interviewer who provided further details on the study procedures and set a comfortable time for the online interview. PwPMS and significant others were informed that a psychologist dedicated to the study was available (scheduled teleconference or telephone call) in the event negative thoughts or distress arose from reading the booklet or from participation in the interview.

The healthcare professionals were selected following convenience sampling. They were contacted by e-mail among those meeting the following inclusion criteria: physicians, psychologists, nurses, social workers, or physiotherapists with expertise in caring for PwPMS and fluency in Italian.

About 2 weeks before the interviews/focus group, participants received the provisional booklet to familiarize with. Participation was voluntary, and no material incentive was given to study participants.

### Data collection

The cognitive interviews and the focus group were held online—to respect COVID-19 restriction measures—between September 2020 and January 2021. All were audio-recorded and transcribed verbatim.

The interviewers (LDP, SV, and AMG) used a pre-planned guide (online supplemental file 4) and prompted PwPMS and significant others to think aloud when answering questions, to explore comprehension and judgments on the ACP booklet items. SV and LDP moderated the focus group using a non-directive style. They asked healthcare professionals to identify unclear or difficult parts/sections and any missing issues. The participants did not know the interviewers and moderators before the study.

### Data analysis

We analyzed the cognitive interviews using the framework method [36] according to an inductive approach considered appropriate for making themes emerge and allowing inter-coder agreement. LDP and SV read the transcriptions extensively and wrote comments and initial thoughts in a memo. They coded the text of the first six interviews line by line independently and produced a provisional list of themes as a framework. The themes were discussed with LG. Then, LDP and SV independently reviewed the themes and, through discussions with LG, renamed them if needed and defined subthemes. LDP applied the framework defined from the analysis of the first interviews to the remaining data. She then extracted the most meaningful narratives from the cognitive interviews to draft the final report, which was checked and amended by SV and LG. MP and LG thematically analyzed the focus group transcript by generating codes describing booklet usability from the healthcare professional perspective [37, 38]. The two researchers independently derived themes and subthemes by gathering codes together. After agreeing on the first themes/subthemes, they met the focus group moderators (LDP and SV) and challenged the provisional themes through discussion. Finally, MP and LG reconfigured the themes and subthemes.

As part of the study, two PwPMS and one significant other selected from the most informative, participated in a second round of interviews to validate revisions to the provisional booklet.

### Reflexivity

The interviewers, moderators, and analysts were experts in qualitative methods and were supervised by a qualitative methodologist (LG). All authors managed to view the data based on interdisciplinary discussions. Therefore, even if their different backgrounds (LDP and MP are researchers and bioethicists, SV is a palliative care physician, LG is a methodologist trained in education, and AMG is a researcher and a psychologist) may have had a role in suggesting interpretations (interpretation bias), the interdisciplinary work on analysis concurred to bracket personal interests or disciplinary assumptions. The interviewers did not have any existing relationship with the interviewees and were external to the work settings of the healthcare professionals.

### Ethical considerations

The ethics committees of each participating center granted formal ethical approvals (Milan, clearance number: 73/2020; Reggio Emilia, clearance number: 2020/0104408; Rome, clearance number: CE/PROG.846). Participants were provided with an information sheet when contacted, and they signed specific, informed consent forms and privacy/confidentiality agreements before data collection.

## Results

### Cognitive interviews

Eleven PwPMS and three significant others (2 women, 2 spouses, and one daughter) agreed to participate in the study. Of these, seven PwPMS and all the significant others were from Northern Italy. One PwPMS withdrew consent after viewing the booklet, which he experienced as too emotionally engaging. None of the participants requested an interview with the psychologist dedicated to the study.

The median (range) age of the 10 interviewed PwPMS was 54 years (43–71); three were women; median Expanded Disability Status Scale score was 6.0 (4.0–8.5); median age at MS diagnosis was 38 years (21–54); median age at progression was 46 years (35–66).

Following the participants’ request, three interviews (2 with PwPMS and one with SO) were conducted on the telephone; one PwPMS was interviewed in the presence of his spouse. The interviews lasted between 36 and 80 min; 10 were held by LDP, two by SV, and one by AMG.

The interviews provided information related to three themes: booklet’s comprehensibility and clarity, content acceptability and emotional impact, and suggestions for improvement. The main quotations for each identified theme are reported in Table 1.

**Table 1:** Quotations from the cognitive interviews with patients and significant others

Theme	Participant	Quotation
Comprehensibility and clarity	Patient Co_08	Yes, the booklet’s title is clear, and it reflects the contents
	Significant other Co_06	I got it… I got the ACP is an agreement between physician and patient about dealing with illness and treatments. However, it isn’t clear if the physician is there with the patient while he/she is reading it
	Significant other Co_06	The description of MS and all the other stuff is quite clear. It is well explained the way we live, on a daily basis
	Patient Co_09	I think that the introduction of the booklet is clear and balanced. The introduction is key to understand the other contents of the booklet and it is balanced
	Patient Co_01	The guidance helps with reading. Its role is clear
	Patient Co_07	I thought it was a self-administered booklet. I believe that the physician role is not so clear. Maybe, this is why it wasn’t easy for me. Only a physician can explain some terms to the patient
	Patient Co_10	The booklet could be shortened because sometimes the same things are repeated
Content acceptability and emotional impact	Significant other Co_02	For example, what does it mean cultural values? It got me in trouble. In our culture, what are the cultural values? In some parts, this tool is far away from the way we are…
	Patient Co_07	I noticed it was translated from New Zealand. This transpires from the language and from how certain topics are treated. [In Italy] we have a rather indirect cultural approach, while this booklet is characterized by rationality, concreteness and extreme clarity. This is not typical of our culture and it is too much. It can hurt!
	Significant other Co_06	It is strong! Here we talk about death. It’s not simple. It is extreme and challenging
	Patient Co_07	These tough topics can evoke challenging emotions. For this reason, I think that we cannot deal with them alone. The presence of the physician is mandatory
	Patient Co_07	It brings up so many things from an emotional point of view. It’s about choices, trust, relationships. They are typical questions of meaning during life, even for people who don’t have a chronic illness These are universal issues, but you have to put them apart to live. Instead, this booklet forces you to write down and put them on paper
	Patient Co_07	Why that sentence about life expectancy? It is written that it could be reduced between 7 and 14 years. I don’t think it’s useful. It seems that I will die soon, so it’s better I start thinking about my death
	Patient Co_09	This booklet forces the physician to be transparent while explaining complicated concepts and helps facilitate the dialogue between the doctor and the patient
	Patient Co_05	I think the images are peaceful… After all, we are talking about death!
Suggestions for improvement	Significant other Co_06	The layout is not very immediate. If you are a schematic person like me, the booklet layout suggests that you write some of the examples reported in the guidance in the left part of the booklet on the right column. Many people will believe that they have to choose between the options listed in the left part of the booklet. You should simplify the layout, maybe using different colors to help on this
	Patient Co_04	I would have used a softer font, but bold or capital letters have been used here instead, it's inappropriate
	Patient Co_01	Sometimes I had problems filling the booklet. Sometimes I struggled to follow along
	Patient Co_07	I strongly dislike the cover image. Why did you choose these cold colors? I don’t understand. It is sad!
	Patient Co_07	In my opinion, images should relieve, while these burden you. They make you feel trapped

#### Comprehensibility and clarity

Participants found the booklet’s title, introduction, and guidance understandable and clear. They reported that the title was consistent with its content and that the guidance embedded in each section was helpful. The examples provided were clear, and the language was plain.

Most participants understood the gradual approach of ACP. However, PwPMS reported that the physician’s role in the ACP process was not openly explained. They also stressed that the booklet needs the presence of a physician to be filled out.

Few PwPMS considered the booklet wordy or redundant.

#### Content acceptability and emotional impact

Participants, particularly PwPMS, reported that the booklet contents were emotionally demanding; some PwPMS found the description of MS quite difficult and suggested removing the sentence on life expectancy. PwPMS found some pictures, including the cover picture, melancholic, and gloomy. Generally, participants considered the booklet helpful in fostering and improving physician–patient communication, by exploring “the unsaid.”

#### Suggestions for improvement

Few participants suggested to improve the layout, which was defined as confusing. One PwPMS suggested using abstract pictures with warm colors. Few participants also asked for better explanations of some unfamiliar words and avoiding repetitions.

### Focus group

Twelve healthcare professionals (7 neurologists, 3 psychologists, one nurse, and one physiotherapist) participated in the focus group, which lasted 105 min. The analysis identified two main themes: booklet content importance and clarity and challenges to ACP implementation. The main quotations for each theme identified are reported in Table 2.

**Table 2:** Quotations from the focus group with health professionals

Theme	Healthcare professional	Quotation
Content importance and clarity	Neurologist Co_04h	It is very understandable. I think it is also exhaustive. Moreover, it drives the patient towards challenging topics gradually
	Psychologist Co_06h	It is an excellent document, I showed it to some of my patients, and they agreed. It seems tailored to this type of patient, and the introduction is smooth, simple
	Neurologist Co_11h	It is too dense. Some topics are reiterated unnecessarily
	Neurologist Co_12h	It should be lightened a bit, and sometimes it is redundant. Is it understandable? Yes, but as one of my colleagues said, especially the second part, it is understandable if someone explains it
	Neurologist Co_11h	Sedation, for example. Here, the topic is quite delicate, strong, and about personal values and beliefs. A patient could think: ‘this is as eliminating a person.’ Of course, the topic is complex; maybe you could handle it a little differently to give a more straightforward message
Challenges to ACP implementation	Neurologist Co_12h	Our patients don’t necessarily want to do ACP and they don't necessarily want to do it at the time. So, in my opinion, this is a point that we need to question. Why do we offer it? Is it a desire that we see in our patients or is it our desire? It’s not necessarily the same for everyone
	Neurologist Co_11h	Your question [when to start the ACP] is a big one. Because a document like this is an elephant in a glass shop Note however that our patients have fears of becoming wheelchair bound ever since they are diagnosed with MS and their concern [about accumulating disability] cannot be ignored. They understand for themselves when the disease is progressing simply by comparing their current situation with that of the previous year. Some just don't want to discuss their disease progression because they don't want their perception confirmed. And we have our own difficulties starting such discussions… So, my answer is that the “when” varies from case to case. There is no general rule
	Psychologist psychotherapist Co_09h	It is necessary to think of a figure with whom the patient can read the booklet because even just the title can be frightening. We are talking about the end of life! If a healthcare professional has not introduced it, or if the ultimate goal of this booklet has not been anticipated, it is likely to be counterproductive
	Neurologist Co_10h	It is comprehensive. Importantly, it is not something the patient can simply pick up, read, and fill out. It is something to be used with guidance. There is a need for guidance and even a discrete amount of time to fill the booklet and understand it
	Psychologist psychotherapist Co_09h	We need to get into the relationship of care and the patient’s awareness of the disease that is co-constructed along the care path. However, it often happens that the awareness of the worsening, especially in people who have secondary progressive form, also corresponds to a lesser ability to conceptualize what is happening While there are fewer cognitive deficits in the primary progressive form, where [ACP] can be implemented, it may be too late from the cognitive point of view in the secondary progressive form

#### Content importance and clarity

Healthcare professionals found that the booklet was relevant to clinical practice, even if they never had any direct experience of ACP. They found the booklet too long and its content redundant. Moreover, some of the proposed topics were identified as unclear, while some subjects were not well explained (e.g., palliative sedation, role of the trustee, futile treatments at EOL).

#### Challenges to ACP implementation

The healthcare professionals found ACP implementation challenging for the difficulty in choosing the right time and their discomfort in talking about EOL issues and choices. For them, the booklet could foster adverse reactions or emotions. Doubts arose about which professional would be responsible for initiating the ACP discussion. Moreover, they convened that patients should use the booklet only together with a healthcare professional. They finally recommended a customized approach to the use of the booklet, tailored to each patient.

### Revision of the booklet and second-round cognitive interviews

Based on the qualitative analysis results, we collected the proposed changes (online supplemental file 5) and revised the booklet (online supplemental file 6) accordingly.

We involved two PwPMS and one significant other in the second-round interviews. They confirmed that the revised booklet was improved in clarity of contents and layout. They reported overall satisfaction with the new pictures. However, they reaffirmed the difficulty in facing the booklet topics and the need for a physician when reading out and completing it.

## Discussion

This multicenter, qualitative study involved intended users (PwPMS, significant others, and healthcare professionals involved in MS care) in the evaluation and refinement of a booklet to be used during the ACP conversations.

Appraisal of the booklet was crucial for improving its comprehensibility and clarity. Both PwPMS and significant others clearly understood that the booklet described ACP as a process for sharing personal decisions about future care, included EOL care. Still, they reported common misunderstandings about the physician’s role in this process. Moreover, they perceived some concepts as unfamiliar. It is worth mentioning that open EOL conversations through ACP are uncommon in Italy. The Italian Law n. 219/2017 “Provisions for informed consent and advance directives” was approved after a fervent public and political debate lasting almost 20 years. The law identifies paths for the affirmation of patient autonomy and a patient-clinician relationship based on reciprocal trust and respect: the patient’s right to consent to or refuse treatment (article 1); pain therapy, dignity at the EOL, and avoidance of unreasonable treatment obstinacy (article 2); advance directives (article 4); and ACP (article 5). Since the law has entered into force, many initiatives have been promoted to facilitate ACP discussions, but they are not clearly structured, and few studies have been conducted to collect data on ACP use in Italy. We chose the New Zealand “My Advance Care Plan & Guide” (online supplemental file 2) because of its structure and embedded guidance, which helps the patient and the healthcare professional navigate along the ACP process, from the identification of patient values to EOL care choices. This tool is part of a New Zealand Ministry of Health initiative to promote consistency in ACP practice and fulfilment of the 1996 Code of Health and Disability Services Consumers’ Rights.

Although PwPMS and significant others appraised the booklet as helpful, they focused more on its emotional impact than on its clarity or comprehensibility. Three types of cognitive bias can explain this finding [39–42]: “Framing bias,” the framing of information (here ACP) which influences the reasoning process; “Projection bias,” when pain, depression, and anger mediate patients’ ability to make consistent treatment choices; and “Present bias,” tending to place excessive weight on the current over the future situation. While the “Framing bias” specifically applies to the present study, where PwPMS and significant others were asked to examine a booklet out of the context for which it is intended to be used (i.e., the ACP process), the other two biases also pertain to the ConCure-SM feasibility trial and were addressed in the trial protocol [30]. Considering the “Present bias,” the training program which is part of the trial specifically focuses on priming neurologists and other healthcare professionals in discussing with patients their future goals of care, including the EOL phase. Considering the “Projection bias,” since a study-related increase in emotional burden cannot be ruled out, PwPMS mood symptoms, assessed through the Hospital Anxiety and Depression Scale, are being recorded at baseline and during follow-up. The independent Data and Safety Monitoring Committee monitors this safety outcome as well as the occurrence of any serious adverse event (admission to psychiatric ward, suicide attempt, death) [30].

Consistently with the most recent literature, healthcare professionals highlighted the common barriers to ACP implementation: hesitance to discuss EOL with patients, fear of causing them distress and loss of hope, lack of knowledge and self-confidence in ACP conversations, and time/space constraints [1, 20–22, 43, 44]. A scoping review on ACP interventions in neurodegenerative disorders found a total of 10 randomized controlled studies, all conducted in the dementia setting [25]. A recently published pilot trial on a nurse-led interview to promote ACP in patients with early dementia showed that the intervention was well received by patients and their significant others. Participants expressed satisfaction with the procedure, especially regarding the opportunity to discuss a sensitive topic with the help of a facilitator [45]. However, only 16 patients were enrolled out of 105 screened; the trial revealed misconceptions about dementia and ACP in patients, significant others, and healthcare professionals, as well as structural and institutional challenges. The authors concluded that “a large scale trial to test a dementia-specific tool of ACP is currently not feasible in Western Switzerland and should be endorsed in a systemic approach of ACP” [45].

Healthcare professionals identified the long trajectory of MS as an additional challenge, with the risk of anticipating too much ACP discussion or deferring to a stage when it is not possible anymore due to patients’ loss of their deliberation capacity. These findings point out the need for training neurologists and other MS healthcare professionals in ACP.

This study has some limitations. Although the cognitive interviews were the most appropriate approach, the topic was challenging, and the interviews often shifted from the cognitive appraisal of the booklet to its emotional impact. Second, data saturation was not discussed (online supplemental file 1). Finally, our findings are specific to the Italian context, and few participants were women; thus, transferability may be limited.

## Conclusions

Acknowledging that our ultimate goal is to provide evidence on the effectiveness of the ConCure-SM intervention, we believe that the present results provide new knowledge on the co-production of an MS-specific ACP intervention and on the challenges envisaged to ACP implementation in Italy.

### Supplementary Information

Below is the link to the electronic supplementary material.

Supplementary file1 (PDF 265 KB)

Supplementary file2 (PDF 3482 KB)

Supplementary file3 (PDF 623 KB)

Supplementary file4 (PDF 542 KB)

Supplementary file5 (PDF 203 KB)

Supplementary file6 (PDF 994 KB)

## Data Availability

Interview and focus group quotations are available in the manuscript, and the interview and focus group guides are provided within the manuscript’s supporting information. Interviews and focus group meeting transcripts cannot be made publicly available as study participants consented to participate with the understanding that their data would remain anonymous and confidential. Further excerpts of the study data are available on request from the Ethics Committee of Fondazione IRCCS Istituto Neurologico Carlo Besta, Milan (institutional contact: comitatoetico@istituto-besta.it).

## Code availability

See “Data availability”.
