# Supplementary material for: User appraisal of a booklet for advance care planning in multiple sclerosis: a multicenter, qualitative Italian study
Source: Neurol Sci. 2023 Oct 10;45(3):1145–54. doi: 10.1007/s10072-023-07087-y (PMC10858142; doi:10.1007/s10072-023-07087-y)
Supplement: Supplementary file 1 — Supplementary file1 (PDF 265 KB) [file 10072_2023_7087_MOESM1_ESM.pdf]

Supplementary File 1

Consolidated criteria for reporting qualitative studies (COREQ):32-item checklist

**No. Item**	**Guide questions/description**	**Reported on Section #**
**Domain 1: Research team and reflexivity**		
Personal Characteristics		
1. Interviewer/facilitator	Which author/s conducted the interview or focus group?	10 interviews were held by LDP, two by SV, and one by AMG.<br>LDP and SV moderated the focus group (Methods – Data collection)
2. Credentials	What were the researcher's credentials? E.g. PhD, MD	AMG: MSc, PhD student<br>LDP: BSc, PhD<br>LG: BSc, PhD<br>MP: BSc, PhD student<br>SV: MD, PhD
3. Occupation	What was their occupation at the time of the study?	AMG: psychologist, PhD student<br>LDP: bioethicist researcher<br>LG: qualitative methods researcher<br>MP: bioethicist, PhD student<br>SV: palliative care clinician and researcher (Methods – Reflexivity)
4. Gender	Was the researcher male or female?	Three were women (MG, LDP, MP), and two men (LG, SV)
5. Experience and training	What experience or training did the researcher have?	All the researchers had expertise in planning, conducting, and analyzing qualitative studies (Methods – Reflexivity)
Relationship with participants		
6. Relationship established	Was a relationship established prior to study commencement?	No relationship established prior to study commencement
7. Participant knowledge of the interviewer	What did the participants know about the researcher? e.g. personal goals, reasons for doing the research	No knowledge (Methods – Data collection)
8. Interviewer characteristics	What characteristics were reported about the inter viewer/facilitator? e.g. bias, assumptions, reasons and interests in the research topic	LDP was co-PI of the study. LDP and SV contributed to protocol devise and they are members of the study's Steering Committee
**Domain 2: study design**		
Theoretical framework		
9. Methodological orientation and Theory	What methodological orientation was stated to underpin the study? e.g. grounded theory, discourse analysis, ethnography, phenomenology, content analysis	The interviews were analyzed using the framework method and the focus group using thematic analysis (Methods – Data analysis)

Participant selection		
10. Sampling	How were participants selected? e.g. purposive, convenience, consecutive, snowball	Purposive sampling (Methods – Research settings and sampling)
11. Method of approach	How were participants approached? e.g. face-to-face, telephone, mail, email	By phone and email (Methods – Research settings and sampling)
12. Sample size	How many participants were in the study?	10 patients, 3 significant others, and 12 healthcare professionals (Results – Cognitive interviews; Results – Focus group)
13. Non-participation	How many people refused to participate or dropped out? Reasons?	One included patient found the booklet emotionally engaging, and refused the interview (Results – Cognitive interviews)
Setting		
14. Setting of data collection	Where was the data collected? e.g. home, clinic, workplace	Online due to the pandemic (Methods – Data collection). Following the participants' request, three interviews were conducted on the telephone (Results – Cognitive interviews)
15. Presence of non-participants	Was anyone else present besides the participants and researchers?	In one case, a patient's wife was present during the interview on the patient's request (Results – Cognitive interviews)
16. Description of sample	What are the important characteristics of the sample? e.g. demographic data, date	All the patients had progressive multiple sclerosis, and were varied in general and clinical characteristics (Results – Cognitive interviews)
Data collection		
17. Interview guide	Were questions, prompts, guides provided by the authors? Was it pilot tested?	Interview and focus group guides are provided in Supplementary file 4). The interview guide wasn't pilot tested; minor changes were made after the first two interviews
18. Repeat interviews	Were repeat interviews carried out? If yes, how many?	Two patients and one significant other selected from the most informative participated in a second round of interviews to validate revisions to the provisional booklet (Methods – Data analysis)
19. Audio/visual recording	Did the research use audio or visual recording to collect the data?	Interviews and the focus group were audio-recorded (Methods – Data collection)
20. Field notes	Were field notes made during and/or after the interview or focus group?	No
21. Duration	What was the duration of the interviews or focus group?	The interviews lasted between 36 and 80 minutes (Results – Cognitive interviews); the focus group lasted 105 minutes (Results – Focus group)
22. Data saturation	Was data saturation discussed?	No (Discussion)

23. Transcripts returned	Were transcripts returned to participants for comment and/or correction?	No, they weren't.
**Domain 3: analysis and findings**		
Data analysis		
24. Number of data coders	How many data coders coded the data?	Four data coders were involved: LDP, LG, MP, SV (Methods – Data analysis)
25. Description of the coding tree	Did authors provide a description of the coding tree?	No
26. Derivation of themes	Were themes identified in advance or derived from the data?	They have been derived from the data (Methods – Data analysis)
27. Software	What software, if applicable, was used to manage the data?	Not applicable
28. Participant checking	Did participants provide feedback on the findings?	No
Reporting		
29. Quotations presented	Were participant quotations presented to illustrate the themes/findings? Was each quotation identified? e.g. participant number	Quotations illustrating the themes and participant code/type are reported in Table 1 (Results – Cognitive interviews) and Table 2 (Results – Focus group)
30. Data and findings consistent	Was there consistency between the data presented and the findings?	Yes (as study authors, we can be biased in the evaluation of this domain)
31. Clarity of major themes	Were major themes clearly presented in the findings?	Yes (as study authors, we can be biased in the evaluation of this domain)
32. Clarity of minor themes	Is there a description of diverse cases or discussion of minor themes?	Not applicable
