# Supplementary material for: User appraisal of a booklet for advance care planning in multiple sclerosis: a multicenter, qualitative Italian study
Source: Neurol Sci. 2023 Oct 10;45(3):1145–54. doi: 10.1007/s10072-023-07087-y (PMC10858142; doi:10.1007/s10072-023-07087-y)
Supplement: Supplementary file 2 — Supplementary file2 (PDF 3482 KB) [file 10072_2023_7087_MOESM2_ESM.pdf]

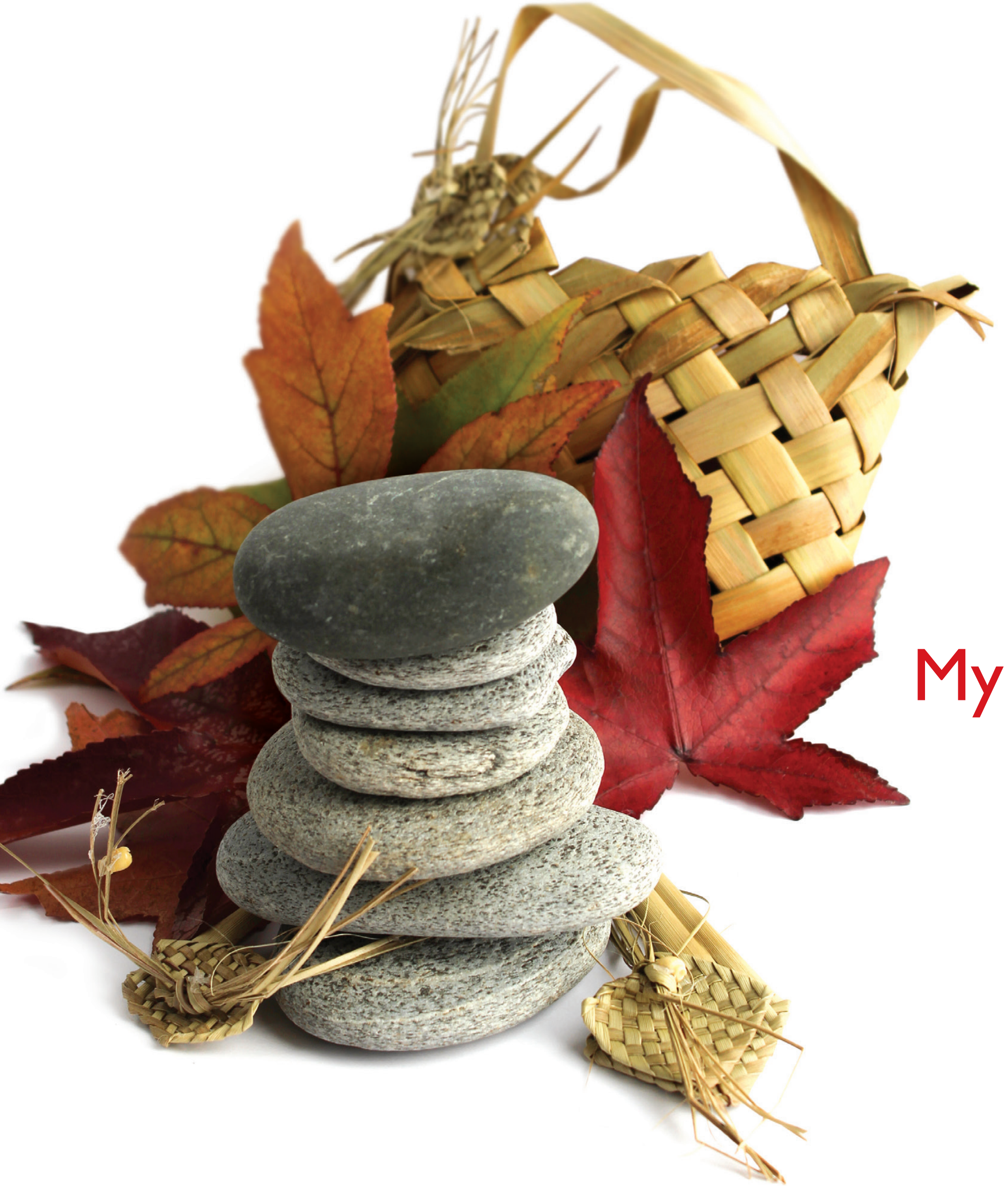

My Advance Care Plan & Guide

Plan the healthcare you want in the future and for the end of your life

Name: ____________________

Date: ____________________

our voice
tō tātou reo
Advance Care Planning

The conversations you have with your whānau and loved ones in thinking about your advance care plan are important, even if you never write down an actual plan.

If you do complete an advance care plan, it needs to be shared with your healthcare team and anyone else you want to have access to it.

It is important your whānau and loved ones know you have a plan and where it is kept.

It is also important you review your plan on a regular basis – maybe every year around your birthday or some other significant date.

Contents

Section 1: My Advance Care Plan	1
Section 2: What matters to me	2
Section 3: Why I'm making an Advance Care Plan	4
Section 4: How I make decisions	6
Section 5: When I am dying	8
Section 6: My treatment and care choices	10
Section 7: After my death	13

The white spaces throughout this booklet are for your choices.

As I work through this plan, these are the questions I have and the things I need to know:

____

____

____

____

____

____

____

____

____

My Advance Care Plan

An advance care plan describes what is important to you as well as the healthcare and treatments you want.

You and your healthcare team can work together to make an advance care plan. This plan will help the healthcare team caring for you, and your whānau and loved ones make decisions about your care **if you can no longer tell them what you want**.

This advance care plan is yours.

You can show it to anyone involved in your healthcare, and give a copy of it to your whānau and loved ones.

You can add to your plan as often as you like and change your decisions at any time. It is important to share any changes you make with the people who have a copy of your plan.

You do not need to complete every section.
Complete only the parts you want.

This guide will help you think and talk about:

- what is important to you now
- how you like to make decisions
- what care and treatment you would like in the future
- what is important to you after your death.

This is my advance care plan and contains my choices.
Please follow this plan if I am unable to tell you what I want.

Last Name:	First Name:
Date of birth:	NHI:
Address:	
Phone:	Mobile:

Or attach patient label if you have one.

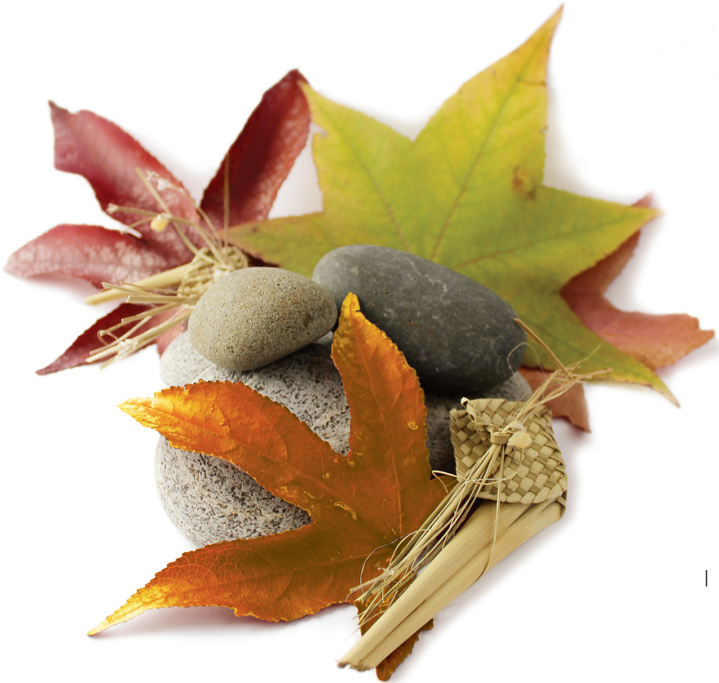

2 What matters to me

Here are some questions to help you work out what matters to you:

- What makes you happy?
- What brings you pleasure and joy?
- How do you like to spend your time?
- What are your hobbies and interests?
- Are there routines you really like?
  for example, how do you like to start or end your day?
- What makes each day meaningful?
- Who do you like spending time with?
- Do you have cultural, religious, spiritual rituals or beliefs ?

Here are some other things that might be important or meaningful to you:

- being able to talk to and be close to people
- being aware of who and where you are
- being able to feel the love and concern of others
- being able to live a life that is meaningful
- being close to a pet
- being able to attend to your spirituality or religion
- being part of your culture
- being able to contribute to society
- being hugged or having your hand held
- being able to walk and/or move around by yourself.

This is what I want my whānau and loved ones and healthcare team to know about who I am and what matters to me:

My cultural, religious and spiritual values, rituals and beliefs:

To honour these beliefs I want my whānau, loved ones and healthcare team to:

2 **What worries me**

Are there things that worry you when you think of your future?

For example, do you worry about:

- how your health might affect your future plans
- how your health might affect your loved ones
- where you will be cared for
- how you will manage pain if it occurs
- being unable to communicate
- being a burden
- going into care
- dying alone
- how your whanau and loved ones will manage without you
- being stuck in bed
- your whānau or loved ones over-riding your wishes
- a clash between traditional and modern cultural ways
- finances?

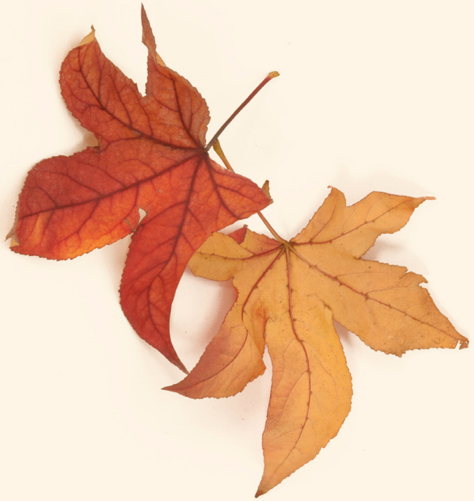

This is what I want my whānau, loved ones and healthcare team to know about what worries me.

I worry about:

○ my loved ones because:

○ suffering. To me this means:

○ not being able to talk or communicate.

○ not doing things such as:

○ other things that worry me are:

○ nothing worries me.

3 Why I'm making an Advance Care Plan

Some things to think about:

- What illnesses have your whānau and family had, and could that happen to you?
- Does your health stop you doing some day-to-day activities?
- Do you have any health conditions you are getting care or treatment for?

To understand what impact your current and future health might have talk to your doctor or healthcare team.

You may need to discuss the following:
Could your illness change:

- how you live your life
- how independent you are
- what you need to plan for?

What might your illness mean for the people who may need to care for you?

This is why I am making my advance care plan:

___

___

___

___

___

◯ I am well.

◯ I am receiving care and treatment for the following:

___

___

___

I understand this may happen to my health in the future:

___

___

___

3 Why I'm making an Advance Care Plan

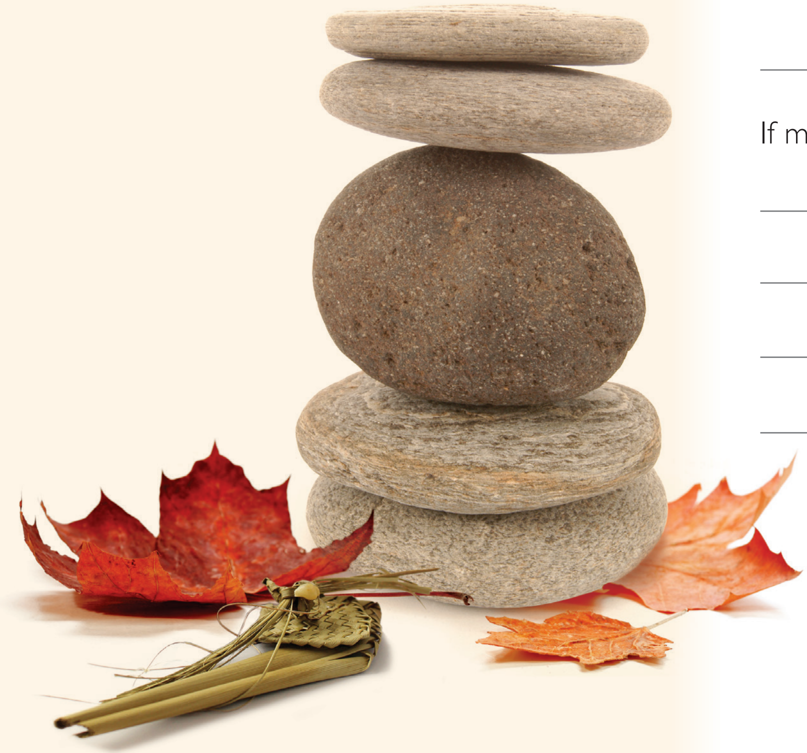

Facing my future makes me think about:

Facing my future makes me feel:

If my time were limited my priorities would be:

4 How I make decisions

Think about the decisions you might need to make about your health.

Think about how you like to make decisions.

Do you need time? Do you like lots of information and options or do you prefer to let others decide?

Sometimes you might be faced with having to make a healthcare decision in a crisis (such as an accident or when you are really sick). This can be made easier for you if you have already thought about how you like to make decisions and who you want involved.

Who can make decisions on your behalf when you are unable to?

If you are too unwell to speak for yourself others will need to help make the decisions for you. Talk to them about what matters to you and what you want *or* don't want to happen while you still can.

If you want a person to have the power to make decisions for you, consider appointing them as your enduring power of attorney for personal care and welfare (EPOA). This means they can be involved in most decisions about your care. This person will not make decisions for you unless you can no longer decide for yourself.

For more information, contact the Citizens Advice Bureau, a solicitor or the Public Trust.

These scales might help you think about how you like to make decisions and how you prefer your medical information is shared. *Mark along the scale what you would want:*

I like to know...

only the basics	○	○	○	○	○	all the details about my condition and my treatment

As doctors treat me, I would like...

my doctors to do what they think best	○	○	○	○	○	to have a say in every decision

If I had an illness that was going to shorten my life, I prefer to...

know my doctor's best estimate for how long I have to live	○	○	○	○	○	not know how quickly it is likely to progress

How involved do you want your loved ones to be?

I want them to do exactly as I have said, even if it makes them uncomfortable	○	○	○	○	○	I want them to do what brings them peace, even if it goes against what I have said

When it comes to sharing information...

I don't want my loved ones to know anything about my health	○	○	○	○	○	I am comfortable with my loved ones knowing everything about my health

4 If I am unable to make decisions

If you appoint an enduring power of attorney for personal care and welfare, include them in any discussions about your future care and treatment options.

Talk them through your advance care plan and give them a copy.

If you do not have an enduring power of attorney, it is a good idea to name someone to help your healthcare team make the best decisions for you.

Talk to this person about what is important to you and how you feel.

For both your enduring power of attorney for personal care and welfare or your nominated person choose someone who:

- knows you well
- cares about what is important to you
- helps you without taking over
- listens to you and is respectful
- will tell people about your wishes and try to make sure they happen.

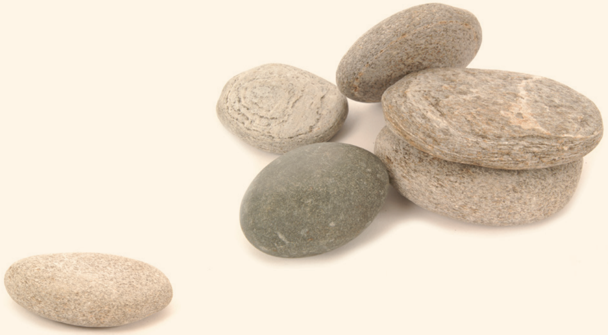

When I am unable to make decisions, I would prefer them to be made like this:

◯ I want my enduring power of attorney for personal care and welfare to make decisions using the information in this advance care plan.

My EPOA's name is: ______________________

Relationship to me: ______________ Phone: ______________

Or

◯ I don't have an enduring power of attorney.

Using the information in this advance care plan, the following person will help my healthcare team make the best decisions for me.

Name: ______________________

Relationship to me: ______________ Phone: ______________

In addition, the following people know me well and understand what is important to me. I would like them included in discussions about my care and treatment.

Name: ______________________

Relationship to me: ______________ Phone: ______________

Name: ______________________

Relationship to me: ______________ Phone: ______________

Name: ______________________

Relationship to me: ______________ Phone: ______________

5 When I am dying

When you are dying you will be made comfortable.

The dying process is different for everyone and will be affected by your age, general health or illnesses and can happen very quickly or it may take several days.

For example, you might need:

- pain-relieving medicines and treatments
- medication to ease breathing difficulties
- medication to manage nausea.

Consider what quality of life may mean to you at this stage of your life:

- being aware and thinking for yourself
- communicating with the people who are important to you
- something else?

What do you think will be important to you when you are dying:

- What would your ideal death look like?
- When you think about dying, what situations worry you?
- Who do you want with you as you die?
- When you are nearing death, what do you want or not want?
- What kind of spiritual care do you want at the end of your life?

As I am dying, my quality of life means:

Other details I would like you to know:

I understand that when I am dying my comfort and dignity will always be looked after.
This will include food and drink if I am able to have them.

In addition, I would like you to:

- ◯ Let the people who are important to me be with me.
- ◯ Take out things, like tubes, that don't add to my comfort.
- ◯ Stop medications and treatments that don't add to my comfort.
- ◯ Attend to my religious, cultural and/or spiritual needs, as I described in section 2.

5 When I am dying

Where would you like to spend your last few weeks or days?

- What would be needed for this to happen?

Who should be contacted when you are dying?

- Where do you keep their contact details?
- Who knows to do this for you?

If your condition meant you couldn't be cared for in your preferred place, where else might you like to be?

What things would be important?
For example, having my loved ones around, maintaining my privacy, etc.

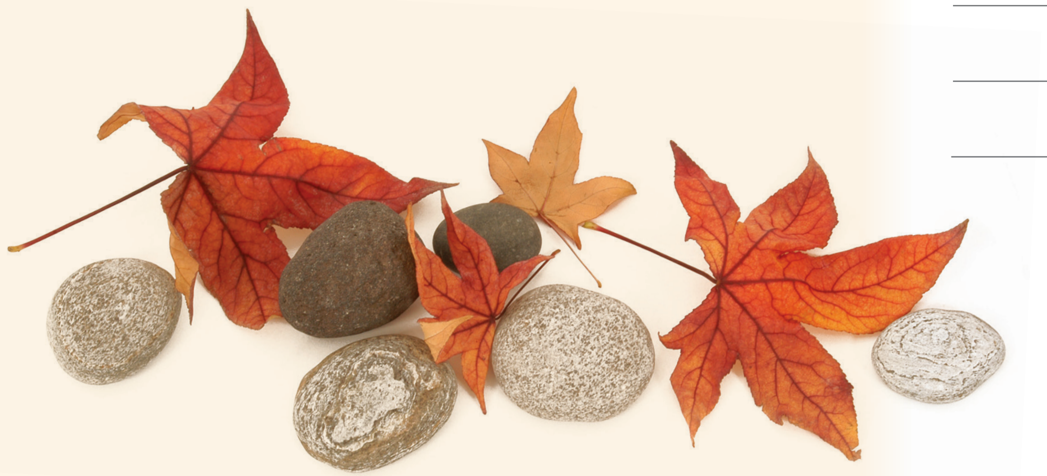

The place I die is important to me: ○ Yes ○ No

When I am dying I would prefer to be cared for:

○ at home, which for me is:

____

____

____

○ in hospital

○ in a hospital level care facility (residential care)

○ in hospice

○ I don't mind where I am cared for

Other details I would like you to know:

____

____

____

____

____

6 My treatment and care choices

This section is best completed with help from a doctor, nurse or specialist.

There are medical procedures that keep you alive or delay death. These may include resuscitation (CPR), life support, getting food and drink through a tube, and kidney dialysis.

Sometimes treatments can be both helpful and harmful. They may keep you alive, but not conscious, or make you a bit better for a short time, but cause you pain.

You need to decide if this is what you want. Your healthcare team will only offer treatments that you will benefit from, this includes the offer of CPR.

Think about what is important to you. For example, quality of life (how good your life is) or quantity of life (how long your life is)?

Are there circumstances in which you would want to stop being kept alive and be made comfortable so you can have a natural death?

If I am seriously ill and I am unable to make decisions for myself, the following best describes the care I would like to receive. I understand this does not require the healthcare team to provide treatments which will not be of benefit to me.

Seriously ill to me means:

___

___

___

Choose only ONE of these five options.

1 I would like my treatment to be aimed at keeping me alive as long as possible. I wish to receive all treatments that the healthcare team think are appropriate to my situation. The exceptions to this would be:

___

If required and appropriate I would want CPR to be attempted:

◯ YES ◯ NO ◯ I will let my doctor decide at the time.

2 I would like my treatment to focus on quality of life. If my health deteriorated I would like to be assessed and given any tests and treatments that may help me to recover and regain my quality of life, but I DO NOT WANT TO BE RESUSCITATED. For me, quality of life is:

___

3 I would like to receive only those treatments which look after my comfort and dignity rather than treatments which try to prolong my life. I DO NOT WANT TO BE RESUSCITATED.

4 I cannot decide at this point. I would like the healthcare team caring for me to make decisions on my behalf at the time, taking into account what matters to me and in close consultation with the people I have listed in Section 4.

5 None of these represent my wishes. What I want is recorded in my Advance Directive on page 11.

I choose Option Number: ______

6 My Advance Directive

If you have treatment and care preferences for specific circumstances or you want an advance directive please write the details below.

An advance directive is a way of choosing beforehand specific treatments you would or would not want in different circumstances if you were no longer able to speak for yourself.

If you can't speak for yourself, it is the responsibility of your healthcare team to apply your advance care plan and any advance directive. When applying the advance directive, they must be confident that you:

(1) fully understood what you were asking for,

(2) were free from influence or duress from someone else, and

(3) meant this to apply to the current situation.

In the following circumstances:	I would like my care to focus on:	I would accept the following treatments:	I would wish to refuse or stop the following treatment:
Example: Severe stroke, unable to recognise anyone	*Example: Allowing a natural death*	*Example: Comfort measures*	*Example: Artificial feeding*

◯ If I have left this section blank, I am happy with the choice I made on the previous page and have no other preferences.

6 Signatures

Your health care team has a responsibility to follow your wishes. Signing this section is optional, but it helps show your healthcare team you fully understand what you are stating. The doctor leading your care will be more confident about using your plan if you sign and date it.

The healthcare professional who helped you complete your plan is also asked to sign it and provide their details.

Remember to share copies of your completed plan with your GP, nurse or specialist, your enduring power of attorney for personal care and welfare or your nominated spokesperson and important whānau and loved ones.

Your rights

Your rights as a patient are set out in the New Zealand Code of Consumer Rights.

- Under the Code advance directives and advance care plans do not need to be formal, written documents.
- They can include any treatments, not just life sustaining treatments.
- Your right to refuse treatment is set out in the New Zealand Bill of Rights Act (Section 11).
- If a healthcare provider violated this right, they would be guilty of a criminal offence.
- A person cannot demand a specific treatment or ask for anything that is illegal.

By signing below, I confirm:

- I understand this is a record of my preferences to guide my healthcare team in providing appropriate care for me when I am unable to speak for myself
- I understand treatments that would not benefit me will not be provided even if I have specifically asked for them.
- I agree that this advance care plan can be in electronic format and will be made available to all healthcare providers caring for me.

Name ____________________

Address ____________________

Phone ________ Signature ________ Date ________

Healthcare professional who assisted me

By signing below the healthcare professional confirms that:

- I am competent at the time I created this advance care plan.
- We discussed my health and the care choices I might face.
- I have made my advance care plan with adequate information.
- I made the choices in my advance care plan voluntarily.

Healthcare Practitioner ____________________

Facility/organisation ____________________

Designation ____________________

Phone ________ Signature ________ Date ________

7 After my death

Have you considered organ and tissue donation?

Donated organs and tissues can help others to live and to have an improved quality of life. For further information go to: Organ Donation New Zealand: www.donor.co.nz

Have you considered leaving your body to medical science?

There are specific processes and forms that need to be completed. For further information contact the Auckland or Otago School of Medicine.

Do you have any body parts that need to be returned to you?

Immediately after death or in the time between death and your funeral, are there any rituals you would like performed?

Is it important where your body is kept?

For your funeral or farewell:

- Do you have preferences for your death announcement?
- Do you have any ideas or preferences for your funeral or farewell?
- Do you already have a prepaid funeral or life celebration plan. If so, with who?
- Are there songs you would like sung or things you wish people to know?

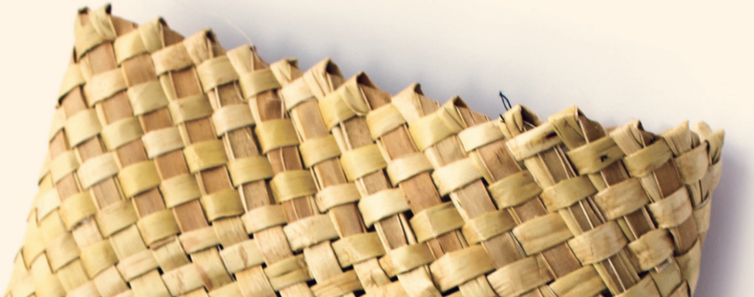

My wishes for organ and tissue donation:

My wishes for caring for my body immediately after death:

After I die I would like to be: ◯ Buried ◯ Cremated

For my funeral or tangi I would like:

____

____

____

____

____

I would like my last resting place to be:

____

This is important to me because:

____

____

◯ I don't mind. I would like the decision to be made by:

7 After my death

Final questions to think about.

Is there anything important you want your whānau and loved ones to know?

Are there any financial records or bank account details that need to be managed?

Have you thought about your social media or Facebook accounts and how these should be managed?

In the years after your death, are there ways you would like to be remembered?

Do you have any final words for your loved ones?

We recommend everyone has a will. If you have a will, who is it with? If you need advice on making a will go to the Citizens Advice Bureau, a solictor or the Public Trust.

© ACP Cooperative 2016.

Kete woven and gifted to the New Zealand ACP Programme by Nga Kaitiaki Kaumatua, Gerontology Nursing Service, Waitemata District Health Board.

Photography by Kara Manson.

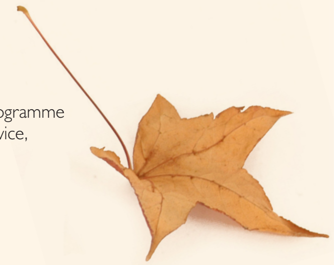

Things I would like my loved ones to know:

My will and other important things can be found:

Document/item	Where it is	Notes
My will		
