# Supplementary material for: User appraisal of a booklet for advance care planning in multiple sclerosis: a multicenter, qualitative Italian study
Source: Neurol Sci. 2023 Oct 10;45(3):1145–54. doi: 10.1007/s10072-023-07087-y (PMC10858142; doi:10.1007/s10072-023-07087-y)
Supplement: Supplementary file 3 — Supplementary file3 (PDF 623 KB) [file 10072_2023_7087_MOESM3_ESM.pdf]

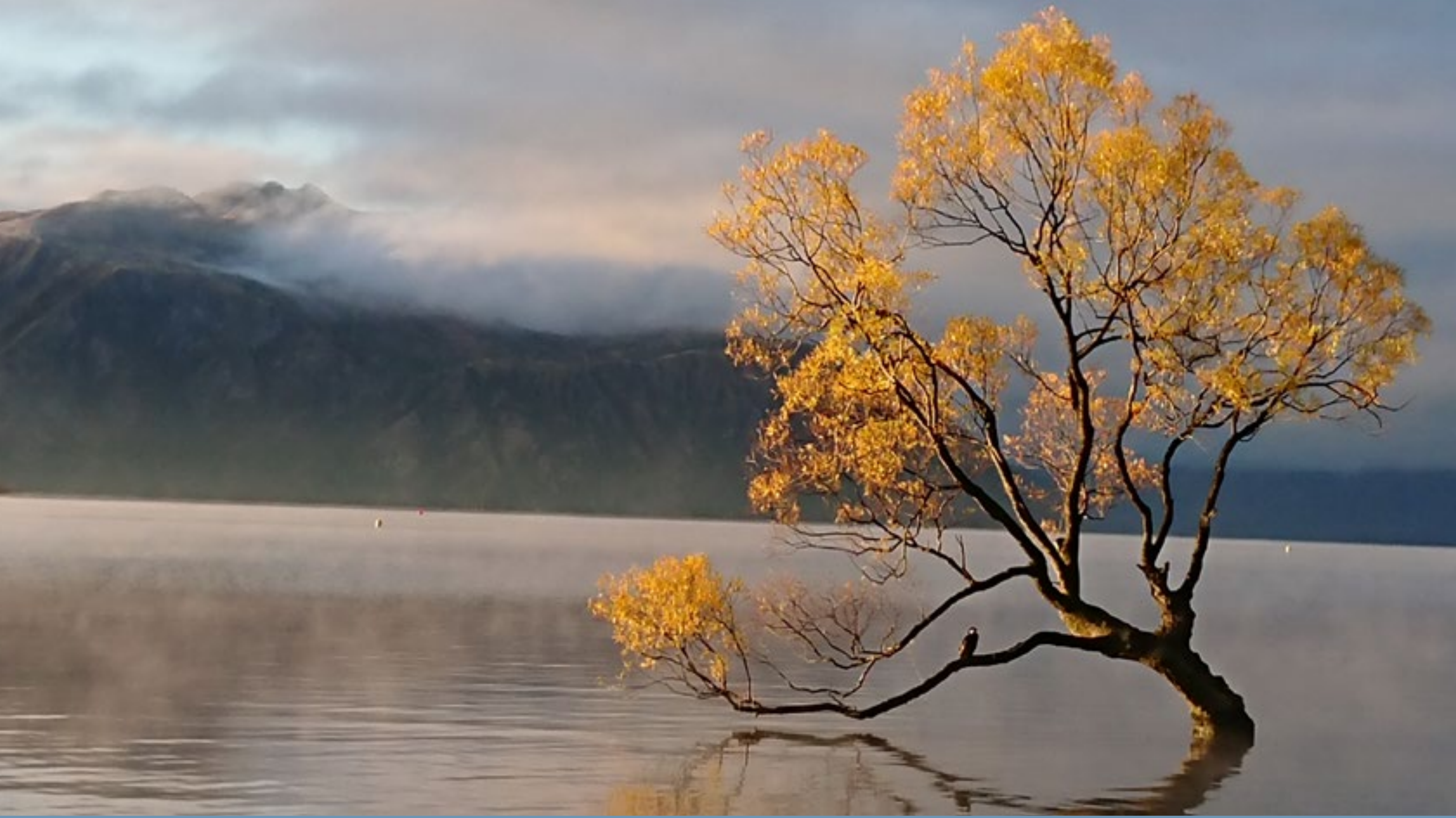

La mia pianificazione condivisa delle cure

Le mie scelte di cura rispetto alla mia salute e al fine vita

È importante quello che tu pensi, ciò in cui credi, quello che vorresti accadesse, o non accadesse, nel corso della tua vita. Se ne parli con i tuoi cari, con le persone importanti per te, con gli operatori sanitari, con chi pensi che ti sarà vicino quando il tuo stato di salute sarà compromesso, sarà più facile per tutti aiutarti nelle decisioni che riguardano la tua vita.

Se però vuoi assicurarti che le tue preferenze vengano rispettate ed abbiano un valore vincolante, devi metterle per iscritto o videoregistrarle. Questo permetterà ai medici di consultarle e di comportarsi in modo da soddisfare i tuoi desideri.

La legge italiana 219/2017 prevede la possibilità di fare delle scelte per situazioni future tramite due differenti modalità. La prima è rivolta a chi sta bene e non ha una malattia progressiva, ma desidera esprimersi rispetto a scelte di cura future, nell'ipotesi in cui perdesse la capacità di decidere o di esprimersi, e si chiama Disposizioni Anticipate di Trattamento (DAT). Le DAT possono essere redatte da qualunque cittadino adulto, adeguatamente informato e capace di decidere. Nelle DAT il cittadino può indicare le sue preferenze e volontà rispetto ai trattamenti sanitari che desidera, o non desidera ricevere, e stabilire se è disposto ad accettare condizioni come l'intubazione, la nutrizione "artificiale", la respirazione meccanica e così via. Le DAT devono essere depositate presso il comune di residenza o presso un notaio. Entrambi si occuperanno di trasmettere il documento ad un registro nazionale consultabile dai medici che entrano in contatto con il cittadino.

La seconda modalità si chiama Pianificazione Condivisa delle Cure (PCC), riguarda chi ha una malattia progressiva, che nel tuo caso è la sclerosi multipla (SM), e viene redatta insieme al proprio medico di fiducia (per esempio il neurologo, il palliativista o il medico di medicina generale). La PCC è un documento che permette al paziente di pianificare le scelte di cura in modo graduale rispetto all'andamento della sua malattia. Essa viene registrata nella cartella clinica o, nelle regioni in cui è attivo, nel fascicolo sanitario elettronico, in modo da poter essere condivisa tra tutti i sanitari che si prendono cura della persona malata. Una copia del documento di PCC rimane al paziente, che potrà conservarla nella sua documentazione sanitaria.

In entrambi i casi la nostra Legge prevede che si possa nominare un fiduciario, ovvero una persona di fiducia che rappresenterà e farà le veci del paziente nelle relazioni con il personale di cura e con le strutture sanitarie, nel caso in cui la persona malata perdesse la capacità di decidere (a causa del peggioramento della malattia, per la comparsa di un evento acuto, o un incidente) e i medici dovessero prendere una decisione importante sulle terapie da iniziare (o non iniziare, o sospendere). In questa evenienza, il fiduciario potrà partecipare alla decisione discutendone con i medici, portando il punto di vista del paziente e le sue preferenze. Si può anche scegliere più di un fiduciario, anche perché non è possibile sapere se la persona identificata in questo compito sarà necessariamente disponibile al momento del bisogno, ma dovrà essere chiaro un ordine di preferenza, per evitare che insorgano contrasti tra i fiduciari rispetto alle scelte. È auspicabile che una copia della PCC sia consegnata anche al fiduciario.

Sia le DAT che la PCC possono essere riviste, ripensate e ridiscusse nel corso del tempo. Questo perché le preferenze e la visione della vita possono cambiare, così come la scelta della persona che si vuole indicare come fiduciario.

Per questo è importante aggiornare regolarmente la PCC, per ripensare alle scelte e ridiscuterle con il medico curante e gli altri professionisti sanitari.

Questo opuscolo è destinato alla persona con SM che desidera redigere la propria PCC. A fianco del **documento di PCC** è presente una **guida** che ha lo scopo di facilitare la compilazione, che deve sempre avvenire attraverso una discussione e condivisione con il proprio medico di fiducia. La guida ti aiuterà a pensare e ad esprimerti su:

- Cosa è importante per te adesso
- Come desideri prendere le decisioni
- Che tipo di assistenza e di cure vorresti per il futuro
- Come vorresti essere assistito alla fine della tua vita

Non devi necessariamente riempire tutti gli spazi di compilazione del **documento di PCC**, ma solo le parti che ti interessano. Ciò che deve essere compilato in ogni parte è la sezione “Firme”.

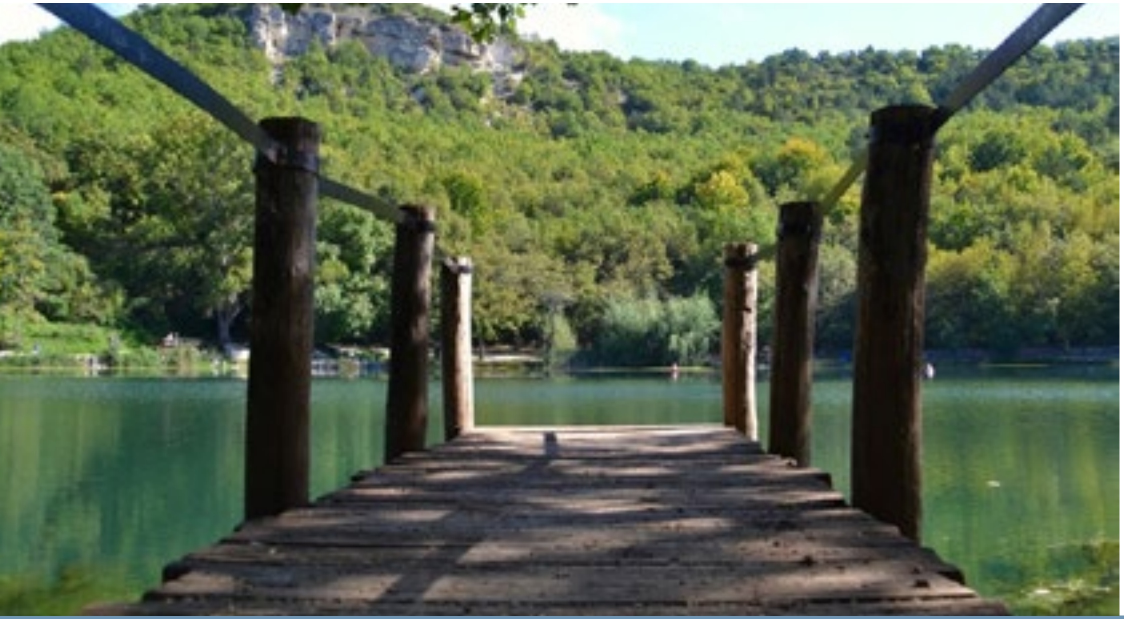

Questo spazio è a tua disposizione per descrivere le tue idee, i tuoi valori, la tua visione della vita e del tuo futuro.

In questo spazio puoi scrivere le tue domande sulle scelte per il futuro, le cure o altre scelte per le quali necessiti di risposte da parte dei medici o di chi si prende cura di te:

La SM è una malattia cronica, caratterizzata da una riduzione variabile dell'aspettativa di vita (tra 7 e 14 anni) rispetto alla popolazione generale, e da un decorso altrettanto variabile. Nella forma progressiva di malattia, i sintomi e le limitazioni funzionali coinvolgono, in modo e con gravità variabile, diversi aspetti, come l'autonomia nei movimenti, la vista, il controllo degli sfinteri, la capacità di alimentarsi, di comunicare, e le funzioni mentali. Questi disturbi possono stabilizzarsi anche per lunghi periodi, permettendo un adattamento personale ed una qualità della vita accettabili, se non soddisfacenti. Ulteriori peggioramenti, come la comparsa di complicanze o di altri problemi di salute e le mutate condizioni familiari che possono verificarsi rendono più difficoltoso questo adattamento continuo. Può accadere di dover condividere la scelta di ricorrere, talvolta in emergenza, a trattamenti di supporto vitale per evitare la morte. Questi trattamenti sono, ad esempio, la tracheostomia (che consente la respirazione facendo passare l'aria direttamente in trachea attraverso un foro chirurgico praticato alla base del collo), oppure la gastrostomia percutanea o PEG (che consente l'alimentazione attraverso un foro chirurgico praticato nell'addome). I trattamenti di supporto vitale possono assicurare anni di vita, tuttavia possono causare ulteriori sofferenze. È utile interrogarsi per tempo sul significato personale di una qualità di vita accettabile.

Lo scopo della PCC è di condividere col proprio medico di fiducia e riportare per iscritto le decisioni rispetto alle scelte terapeutiche ed assistenziali che potranno essere necessarie nel corso della malattia.

Essa costituisce uno strumento vincolante rispetto a queste specifiche decisioni ed aiuterà i tuoi curanti e i tuoi cari a prendere le decisioni qualora tu non potessi più esprimerle.

Questa PCC è tua, ma potrai modificarla in accordo con il tuo medico di fiducia ogni volta che vorrai, avendo cura di condividere il nuovo piano anche con il tuo fiduciario che potrà avere la possibilità di confermare il suo ruolo o meno, a seconda delle indicazioni e preferenze che indicherai.

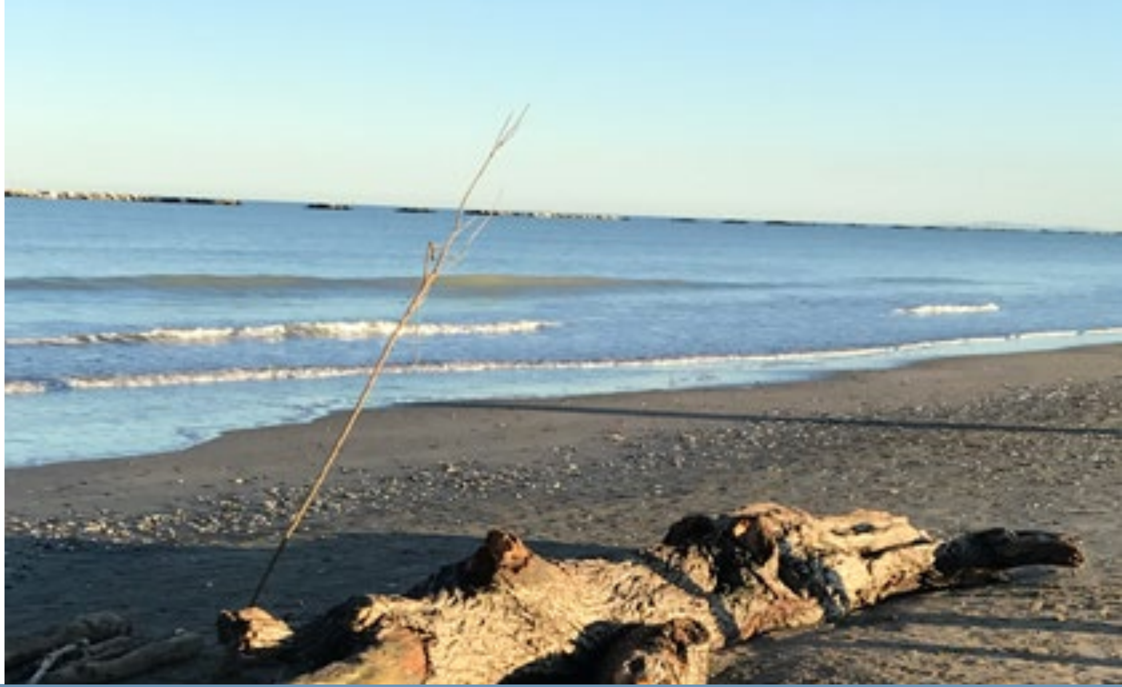

Contenuti:

1. La mia pianificazione condivisa delle cure
2. Cosa è importante per me
3. Cosa mi preoccupa
4. Perché voglio fare una ‘Pianificazione Condivisa della Cure
5. Come prendo le decisioni
6. Se non fossi più in grado di decidere: il mio fiduciario
7. Pensando alla fine della mia vita
8. Le mie scelte di cura
9. Firme
10. Abbreviazioni

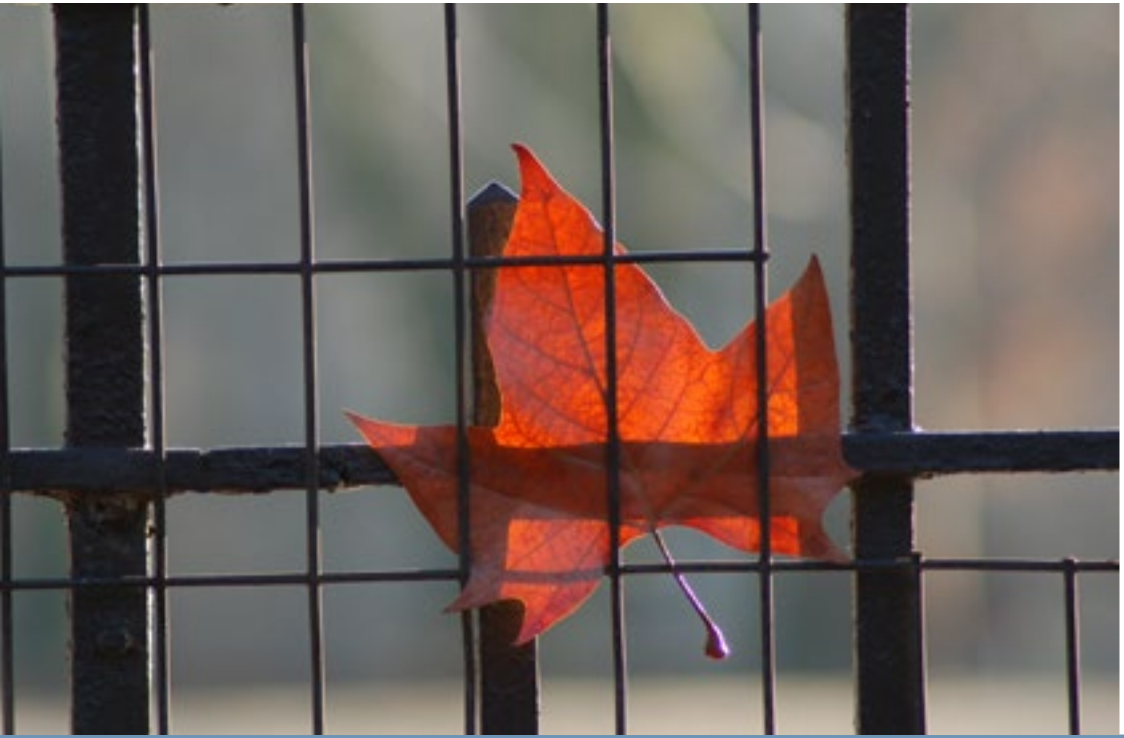

1. La mia pianificazione condivisa delle cure

Questa è la mia Pianificazione Condivisa delle Cure e contiene le mie scelte.

Per favore, seguitela qualora non fossi più in grado di esprimere quello che desidero:

Nome **Cognome**

Nato/a il: **a:**

Indirizzo:

Telefono:

E-mail

2. Cosa è importante per me

Alcune domande che possono aiutarti a definire cosa sia importante per te:

- Cosa ti rende felice?
- Cosa ti reca piacere e gioia?
- Che cosa ti piace fare?
- Quali sono i tuoi hobby e i tuoi interessi?
- Ci sono delle abitudini alle quali sei affezionato?
- Che cosa dà senso alla tua giornata?
- Con chi ti piace trascorrere il tempo?
- Hai principi spirituali, religiosi, o riti che sono importanti per la tua vita?

Ecco alcune altre cose che potrebbero essere importanti o significative per te:

- Parlare e stare vicino alle persone
- Renderti conto di chi sei e dove ti trovi
- Sentire l'amore e l'affetto degli altri
- Vivere esperienze significative
- Avere vicino il cane o l'animale di compagnia
- Partecipare al culto della mia religione
- Sentirti attivo culturalmente
- Contribuire al bene della società
- Sentire che qualcuno ti abbraccia e ti tiene per mano
- Mantenere il più possibile l'autonomia
- Avere momenti di intimità o sessualità

Questo è ciò che voglio che i miei curanti ed i miei cari sappiano di me, e di cosa è importante per me:

Questi sono i valori culturali, spirituali, religiosi e i riti importanti per me:

Per onorare questi valori desidero che i miei curanti e i miei cari:

3. Cosa mi preoccupa

Ci sono cose che ti preoccupano quando pensi al tuo futuro?

Per esempio, ti preoccupi quando pensi:

- Che la tua salute potrà compromettere le tue scelte
- Che la tua salute potrà causare problemi ai tuoi cari
- Dove sarai assistito in futuro
- Di provare dolore o sofferenza
- Di non essere più in grado di comunicare
- Di perdere la capacità di ragionare
- Di essere di peso per gli altri
- Di venire ricoverato in struttura
- Di morire da solo
- Di come le persone che ami possano andare avanti senza di te
- Di rimanere bloccato in un letto
- Che le tue scelte non siano rispettate
- Che i tuoi valori non siano considerati
- Di avere problemi economici

Questo è ciò che voglio che i miei curanti e i miei cari sappiano rispetto a ciò che mi preoccupa:

Segna le caselle corrispondenti

Soffrire. La sofferenza per me significa:

Non poter comunicare, ad esempio:

Non poter far cose, ad esempio:

Mi preoccupo per i miei cari perché:

Altre cose che mi preoccupano:

4. Perché voglio fare una Pianificazione Condivisa delle Cure

Alcune cose a cui pensare:

- Come è stato l'andamento della tua SM e della tua salute in generale nell'ultimo anno?
- Il tuo stato di salute ti limita fortemente in attività che sono importanti per te?
- Sei aiutato e sostenuto da familiari e più in generale da persone care?
- Sei di aiuto e sostegno a familiari e persone care?

Per comprendere meglio che impatto potrà avere il tuo stato di salute sul tuo futuro, parlane con i professionisti sanitari che si prendono cura di te.

Per esempio, potresti chiedere loro: Se la mia SM dovesse peggiorare...

- Che livello di indipendenza potrò avere?
- Cosa è bene/giusto pianificare ora?
- Cosa accadrà al mio corpo e alla mia mente?
- Che impatto potrebbe avere il mio stato di salute sulle persone che si prendono cura di me?

Ecco perché voglio fare una PCC:

Se penso al mio futuro mi viene in mente:

Se penso al mio futuro mi sento:

Se il tempo davanti a me fosse breve allora vorrei:

5. Come prendo le decisioni

Pensa alle decisioni che potresti dover prendere nel corso della malattia.

Pensa a come sei abituato a prendere le decisioni.

Hai bisogno di tempo? Ti piace essere molto informato sulle possibilità di scelta, o preferisci che siano altri a decidere per te?

Hai mai pensato che nella vita possano verificarsi eventi improvvisi, come incidenti o eventi acuti, in cui debbano essere prese rapidamente delle decisioni importanti?

Chi vorresti che decidesse per te, se tu non fossi in grado di farlo?

Ricorda che, qualora non fossi più in grado di esprimerti, altri dovranno decidere per te. Prenditi dunque del tempo per pensare e per parlare di questo con le persone che ti sono vicine.

Se decidi di nominare una persona come tuo fiduciario, perché pensi che possa rappresentare adeguatamente il tuo punto di vista nelle decisioni che riguardano la tua salute, potrebbe essere il momento giusto per farlo. Potrai revocare questa scelta in ogni momento. Il tuo fiduciario deciderà per te solo in caso tu non possa esprimere la tua preferenza.

Rispondendo a ciascuna delle affermazioni riportate di seguito potrai chiarire meglio le tue preferenze relative alle scelte di cura che ti riguardano.

Segna la casella che più corrisponde alla tua preferenza

Voglio avere...

Solo le informazioni strettamente necessarie	Tutti i dettagli sulla mia malattia e le terapie

Voglio che i miei curanti...

Facciano quello che pensano sia meglio per me	Mi consentano di dire la mia in ogni circostanza

Se la mia SM raggiungesse una fase avanzata vorrei...

Sapere quanto mi resta da vivere	Non sapere quanto mi resta da vivere

Voglio che i miei cari…

Decidano rispettando esattamente la mia volontà, anche se questo li facesse stare male	Prendano la decisione che li faccia sentire in pace, anche se dovesse essere contraria alla mia volontà

Voglio che i miei cari…

Non sappiano nulla sul mio stato di salute	Ricevano ogni informazione sul mio stato di salute

6. Se non fossi più in grado di decidere: il mio fiduciario

Se hai deciso di nominare un fiduciario, devi coinvolgerlo nelle tue scelte future.

Parla con lui, o con lei, del tuo piano di cure e consegna al fiduciario una copia del documento di PCC, dopo che l'avrai compilato.

Se non hai ancora deciso di nominare un fiduciario, prova a pensare se non sia il caso di farlo ora.

Se devi scegliere una persona, o più persone, che dovranno decidere per la tua salute nel momento in cui tu non fossi più in grado di farlo, scegli qualcuno che:

- Ti conosca bene
- Si preoccupi di cosa è importante per te
- Sia disponibile a parlare di questi aspetti con te
- Ti ascolti e sia rispettoso
- Sia disposto a difendere le tue volontà affinché vengano esaudite.

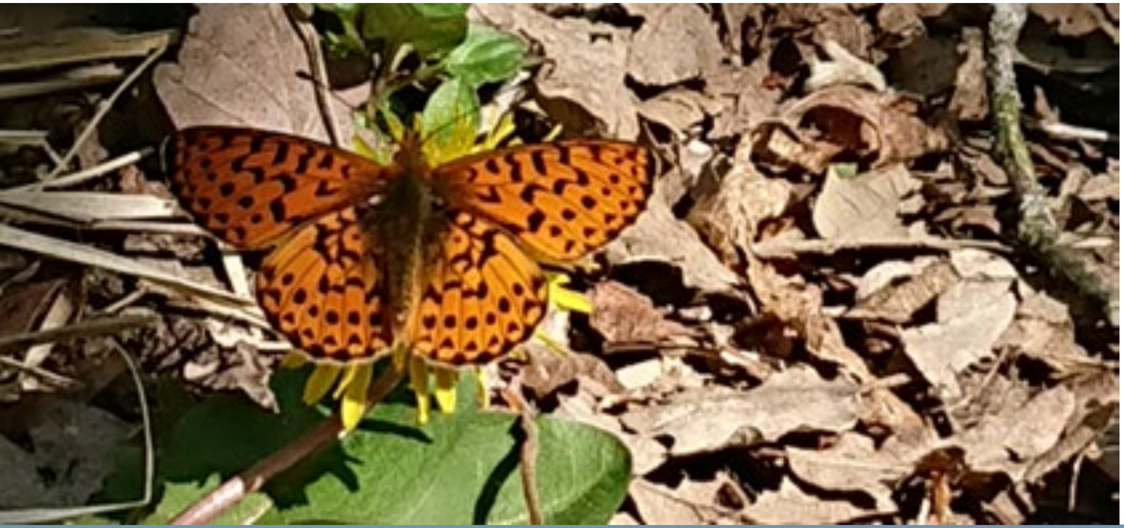

Se perdessi la capacità di decidere, vorrei che:

Segna la casella che corrisponde alla tua preferenza

Le decisioni riguardanti le mie cure future venissero concordate con il mio fiduciario di seguito indicato:

Nome e Cognome

Indirizzo

Telefono e-mail

Se il mio fiduciario fosse impossibilitato a svolgere il suo ruolo, indico come seconda, terza persona di fiducia:

Nome e Cognome

Telefono e-mail

Nome e Cognome

Telefono e-mail

Oppure:

Non ho scelto un fiduciario.

Vorrei inoltre che la persona di seguito indicata sia comunque informata dai sanitari che prenderanno decisioni sulle mie cure future in base alle indicazioni contenute in questo documento ed in funzione del mio migliore interesse.

Nome e Cognome

Indirizzo

Telefono e-mail

7. Pensando alla fine della mia vita

Morire è parte del vivere, ma ci preoccupa e spaventa. È desiderabile che la fine della vita avvenga nel rispetto della propria dignità e autonomia, in un luogo adeguato e possibilmente di nostra scelta, in presenza delle persone a noi care, se lo vogliamo, e limitando ogni tipo di sofferenza. Non esiste un percorso uguale per tutti alla fine della vita, esso infatti può essere influenzato dall'età, dalle malattie di cui soffriamo e da altre circostanze. In questa fase, potrebbe essere necessario ricevere farmaci e trattamenti con l'obiettivo di controllare sintomi che possono presentarsi quali dolore, mancanza di fiato, nausea, ansia, agitazione. Nei rari casi nei quali la sofferenza non fosse gestibile con terapie ordinarie potrebbe essere indicata una sedazione palliativa profonda, ovvero un trattamento che annulla gradualmente la coscienza, con lo scopo di ridurre la sofferenza sino al sopraggiungere della morte (la sedazione palliativa profonda infatti non anticipa né procrastina il momento della morte).

Pensando a cosa significhi per te mantenere una buona qualità della vita, in questa fase cosa credi che sarebbe importante?

- Restare vigile e mantenere il controllo il più a lungo possibile
- Non sentire alcuna sofferenza anche a costo di essere sonnolento o addormentato
- Avere accanto chi amo
- Stare da solo

Dovendo pensare alla fine della tua vita:

- Quale sarebbe la tua morte ideale?
- Pensando alla morte ed al morire, cosa ti preoccupa di più?
- Chi vorresti avere accanto?
- Che tipo di assistenza spirituale o religiosa vorresti?
- In prossimità della morte, cosa vorresti e cosa non vorresti?

Per me una buona qualità della vita in prossimità della morte significa:

Vorrei anche aggiungere:

Quando starò morendo desidero essere curato e accudito nel rispetto della mia persona e della mia dignità. Inoltre desidero:

Segna la casella che corrisponde a ciò che desideri

- Che vengano rimossi tubi ed altri presidi che possano ostacolare il contatto con le persone che mi sono care
- Che vengano interrotti trattamenti non più utili
- Avere un sostegno spirituale o religioso

7. Pensando alla fine della mia vita

Dove vorresti trascorrere le tue ultime settimane o giorni?

- Cosa ritieni necessario affinché questo possa avvenire?

Chi dovrà essere informato del fatto che stai per morire?

- Dove conservi i contatti (nome, telefono) di queste persone?
- C'è qualcuno che potrà contattarle?

Nel caso non fosse possibile soddisfare la tua scelta sul luogo dove morire, hai altre preferenze da esprimere?

Quali altre cose sarebbero importanti per te? (Per esempio, mantenere la tua privacy, ascoltare una musica particolare, poter vedere alcune persone significative, ecc.)

Il luogo nel quale morire è importante per me:

Segna la casella che corrisponde alla tua preferenza

Sì **No**

Quando starò morendo vorrei essere assistito:

A casa, che per me significa:

In ospedale

In una struttura (comunità, casa di riposo)

In hospice

Non è rilevante il luogo dove sarò assistito

Altri aspetti che vorrei venissero considerati:

8. Le mie scelte di cura

Questa parte del documento va compilata con l'aiuto del tuo medico di fiducia.

I trattamenti di supporto vitale possono mantenerti in vita nelle stesse condizioni in cui ti trovi ora. Altre volte essi possono consentire condizioni di vita per te inedite e difficili da immaginare, o risultare fastidiosi o dolorosi. Tra questi trattamenti vi sono l'idratazione/nutrizione 'artificiale' (per sondino naso-gastrico, PEG, per via parenterale/endovenosa), la rianimazione cardiopolmonare (RCP), la ventilazione meccanica con o senza tracheostomia, la dialisi. I trattamenti di supporto vitale in sé non sono né buoni né cattivi, dipende da come e quando vengono utilizzati. È importante, inoltre, ricordare che questi trattamenti non devono essere considerati irreversibili e che si può tornare indietro anche in queste scelte.

Puoi decidere se ricevere, o meno, questi trattamenti. I tuoi curanti ti proporranno solo trattamenti utili per la tua condizione, come per esempio la RCP, che potrebbe riattivare la funzione del cuore o dei polmoni. In tal caso sei chiamato a decidere se vuoi che venga fatta o meno.

Pensa a cosa è importante per te. Per esempio, la qualità della tua vita (non soffrire) o la durata della tua vita (poter vivere il più a lungo possibile). La tua PCC serve in particolare nelle condizioni di emergenza, ove tu non sia in grado di prendere delle decisioni per facilitare i curanti a mettere in atto o meno, trattamenti nel tuo miglior interesse. Trattamenti appropriati sul piano strettamente tecnico, potrebbero infatti essere inappropriati alla luce delle tue preferenze.

Ci sono circostanze nelle quali non vorresti essere mantenuto in vita e preferiresti non iniziare o sospendere terapie di supporto?

Se mi trovassi in condizioni di estrema gravità, in pericolo di vita ed incapace di decidere per me, ciò che segue descrive al meglio le mie preferenze di cura. Sono consapevole di non poter pretendere trattamenti che i medici giudichino inappropriati per le mie condizioni. Estrema gravità per me significa:

Scrivi nel riquadro in fondo alla pagina il numero corrispondente alla tua scelta di cura e, dove applicabile, indica con un segno i trattamenti specifici (punto 2).

1 Vorrei ricevere **tutti i trattamenti disponibili** ritenuti necessari e appropriati dai medici che mi cureranno, per mantenermi in vita il più a lungo possibile.

2 Vorrei ricevere **solo quei trattamenti** mirati non solo a prolungare, ma anche a preservare una qualità di vita ancora accettabile per me.

Nello specifico, accetto di ricevere i seguenti trattamenti:

idratazione/nutrizione per sondino naso-gastrico
PEG
idratazione/nutrizione parenterale/endovenosa
rianimazione cardiopolmonare
ventilazione meccanica senza tracheostomia
ventilazione meccanica con tracheostomia
dialisi

3 Vorrei ricevere solo le cure mirate al **controllo dei sintomi e al mio comfort**, nel rispetto della mia dignità. Non voglio alcun trattamento finalizzato solo a prolungare la mia vita.

4 Non sono in grado di decidere adesso. **Delego i medici** che mi cureranno a prendere le decisioni migliori per me, tenendo in considerazione il parere delle persone che ho indicato nella sezione 6.

Ho scelto l'opzione numero:

Ho già redatto le mie **Disposizioni Anticipate di Trattamento**, depositate presso il comune

di in Data

e reperibili presso il Registro Nazionale DAT.

Questo documento:

Aggiorna le mie DAT Conferma le mie DAT

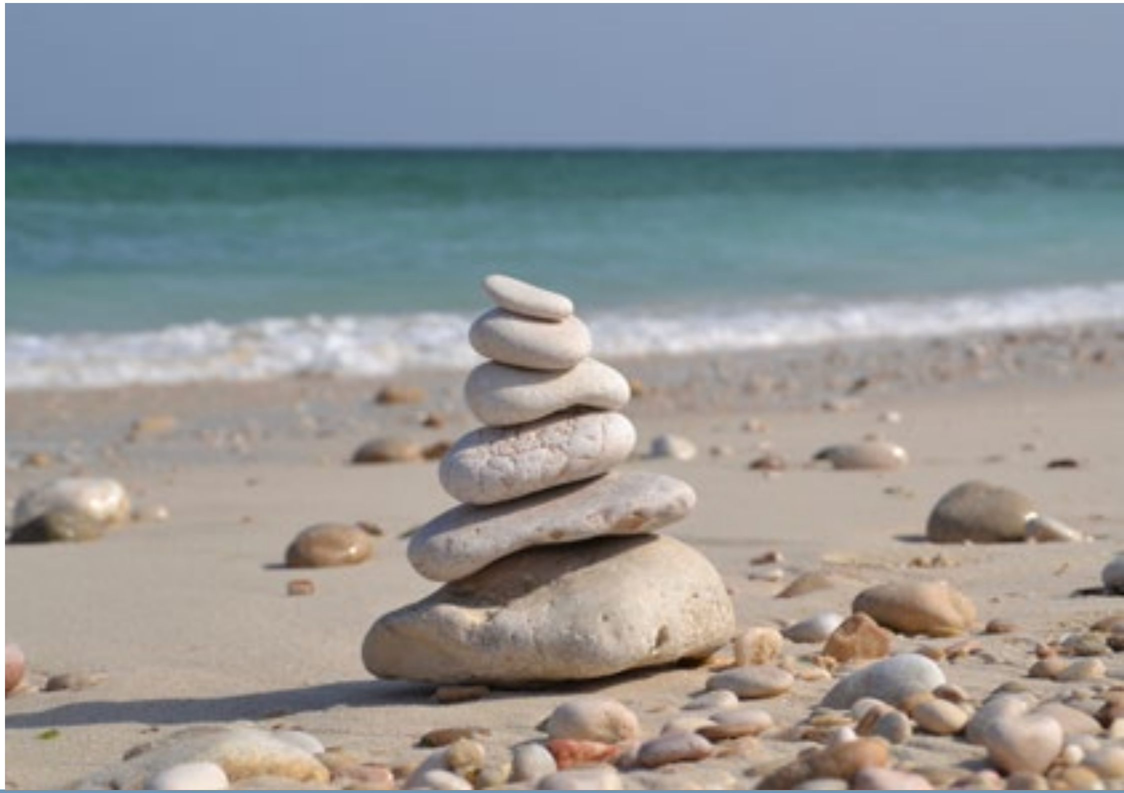

9. Firme

La firma di questo documento è necessaria affinché esso sia ritenuto valido e sia applicato. Se non puoi firmare, è sufficiente una videoregistrazione in cui i sanitari leggeranno le sezioni 6, 7 e 8 del documento e registreranno le tue scelte.

Se hai nominato un fiduciario, è necessaria anche la sua firma.

Anche il tuo medico di fiducia, ed eventuali altri professionisti sanitari che ti hanno in cura dovrebbero firmarlo, perché questo garantisce che la PCC è avvenuta in modo informato e condiviso.

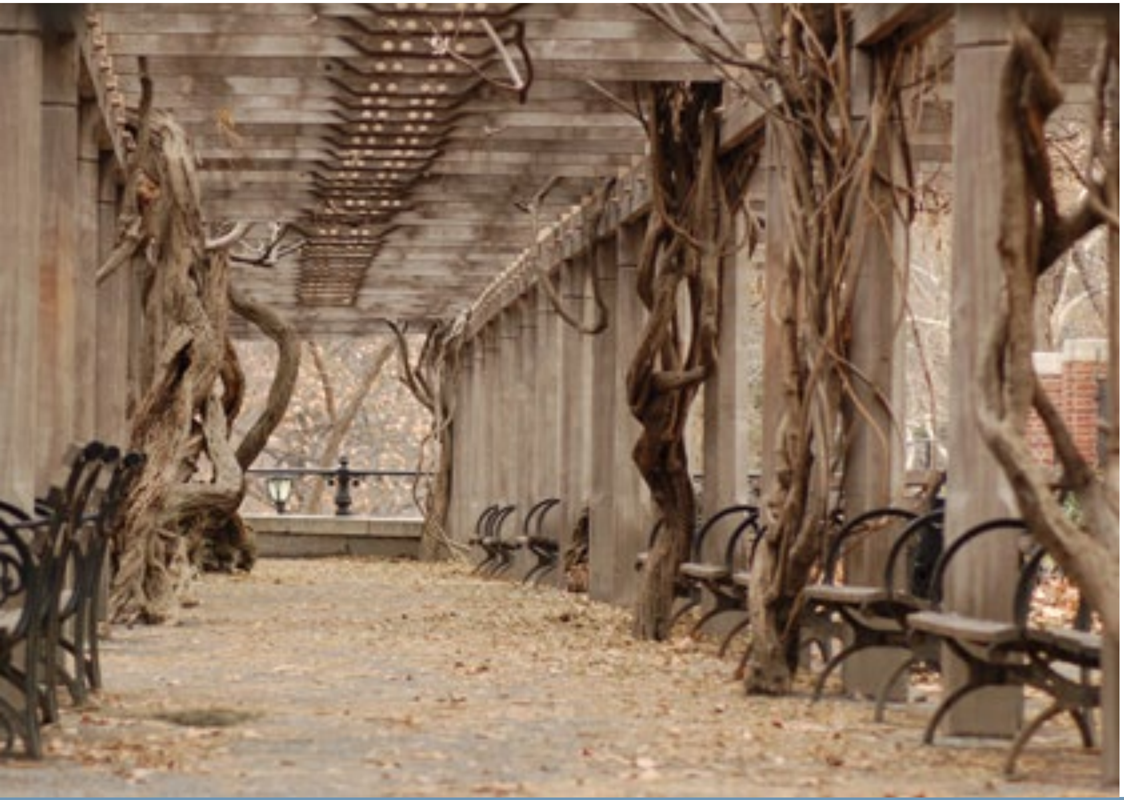

Firmando questo documento io confermo:

1. Di avere compreso la finalità dello stesso e che esso rispecchia le mie volontà
2. Di averlo compilato in piena libertà e dopo essere stato adeguatamente informato
3. Di acconsentire alla conservazione delle informazioni nei registri, nelle cartelle cliniche e nei fascicoli elettronici previsti, secondo la normativa sulla privacy (Regolamento UE 2016/679) e relativa normativa italiana di adeguamento (D.Lgs. n. 196 del 30 Giugno 2003, così come modificato dal D.Lgs. n. 101 del 10 Agosto 2018).

Nome e Cognome

Indirizzo

Telefono e-mail

Data Firma

Ho scelto come fiduciario:

Nome e Cognome

Indirizzo

Telefono e-mail

Data Firma

Non ho scelto un fiduciario

Ho condiviso con il mio medico di fiducia questo documento:

Dr

Telefono e-mail

Data Firma

E, dove applicabile, con il professionista sanitario:

Dr

Telefono e-mail

Data Firma

10. Abbreviazioni

DAT: Disposizioni anticipate di trattamento

PCC: Pianificazione condivisa delle cure

PEG: Gastrostomia percutanea endoscopica

RCP: Rianimazione cardiopolmonare

SM: Sclerosi multipla

Autori: Michela Bruzzone[1], Marta Cascioli[2], Ludovica De Panfilis[3], Andrea Giordano[4], Maria Grazia Grasso[5], Alessandra Lugaresi[6], Luisa Motti[7], Emanuela Pelle[8], Eugenio Pucci[9], Alessandra Solari[4], Claudio Solaro[10], Simone Veronese[8]

1. Associazione Italiana Sclerosi Multipla, Genova
2. Hospice 'La Torre sul Colle', Spoleto (PG), Azienda USL Umbria 2
3. Azienda USL-IRCCS di Reggio Emilia, Reggio Emilia
4. Fondazione IRCCS Istituto Neurologico Carlo Besta, Milano
5. Fondazione Santa Lucia IRCCS, Roma
6. IRCCS Istituto delle Scienze Neurologiche di Bologna; Università di Bologna
7. Hospice 'Casa Madonna dell'Uliveto', Albinea (RE)
8. Fondazione F.A.R.O. Onlus, Torino
9. UOC Neurologia, ASUR Marche, AV4, Fermo
10. CRRF M. L. Novarese, Moncrivello (VC)

Questo opuscolo fa parte del Progetto ConCure-SM, è la traduzione e adattamento di uno strumento di PCC prodotto dalla National ACP programme for New Zealand, 021 928581 Health Quality & Safety Commission.

Realizzazione grafica e stampa resi possibili grazie al contributo dell'Associazione Marchigiana Sclerosi Multipla e altre Malattie Neurologiche.

Foto di copertina, p. 8, 18, 28, Nicola Lugaresi. Foto p. 4 e 27 Chiara Uncini.
