# Supplementary material for: User appraisal of a booklet for advance care planning in multiple sclerosis: a multicenter, qualitative Italian study
Source: Neurol Sci. 2023 Oct 10;45(3):1145–54. doi: 10.1007/s10072-023-07087-y (PMC10858142; doi:10.1007/s10072-023-07087-y)
Supplement: Supplementary file 4 — Supplementary file4 (PDF 542 KB) [file 10072_2023_7087_MOESM4_ESM.pdf]

Supplementary File 4

Guide of the cognitive interview

Opening

- Interviewer introduces him/herself and explains the duration of interview, its recording, and data protection issues.
- He/she explains the purpose of the booklet and its development.
- He/she then explains the purpose of the interview: 'We will review each part of the booklet you have received, starting with what you describe as unclear. After reading, try to think aloud to answer my questions. I apologize if the questions are repetitive or the interview becomes boring. In this study, we are not much focused on what you wrote/would write in the fillable sections of the booklet. Instead, we are interested in what you think of the contents, and how you formed your answers to any given questions. You can tell me any thoughts or opinions, whatever comes to your mind'.
- Looking at the cover - the writing and the pictues - what comes to your mind? How did you find the layout?
- What parts did you point out/find unclear? May I ask you to point me to the first one?

FOR <u>EACH</u> SENTENCE/SECTION/QUESTION [*], THE FIVE ITEMS BELOW ARE REPEATED

1. **Comprehension**

- What does [*] mean to you, in your own words?
    - What does the word ____ mean to you?
    - What is your understanding of [*]?
    - Can you explain [*] to me?
- How easy or difficult was to understand [*]?
- (If applicable) what and how would you change [*]?

2. **Retrieval**

- What did you think while answering [*]?
    - Was it easy or difficult?
    - When you read [*], what were you thinking about?

3. **Judgment**

- How confident are you about the answer you gave to [*]?
    - In general, what was the purpose of [*]?
    - How confident are you that you properly understood the purpose of [*]?

4. **Answers/Response options**

- Do all the response options make sense for [*]?
    - Was it difficult or easy to choose a response from the options provided?

    - How did you select an answer to [*]?
- Is there anything else you would like to say about [*]?

5. Other issues

- Have you any other issues regarding [*]?

Close of the interview

- What is your general opinion about the booklet?
- How could it be improved?
    - Are there any contents you would leave out of this booklet?
    - Are there any contents you would like to add to this booklet?
- Did you find any contents indelicate or somehow inappropriate?
- Is there anything else you would like to say about the booklet as a whole?
- Interviewer thanks the participant.

Guide of the focus group

Opening

- Moderator introduces himself, explain his role during the meeting.
- He then introduces the co-moderator and her role during the meeting.

Purpose of the focus group topic

- Expected duration, audio-recording, data protection
- Ground rules of the meeting (all responses are important—try not to interrupt or talk over each other; all responses are valid—there are no right or wrong answers; try to stay on topic so that all the questions are covered)
- Brief explanation of booklet purpose and development

Participants introduce themselves

First round question

- Comprehensibility: is there anything in the booklet that seemed unclear or difficult to understand?

Second round question

- If you plan to use the booklet in your practice, which sections do you find most difficult for patients/significant others to understand/manage?

Third round question

- What are the sections of the booklet that you think are difficult to deal while using it with your patients/significant others?

Close of the meeting

- Moderator summarizes the main discussion points, and ask participants if they have any additional thoughts to share.
- He thanks the group for participating and reminds them that in a few days a written report will be produced, and sent to each participant via e-mail for validation.
