# Supplementary material for: User appraisal of a booklet for advance care planning in multiple sclerosis: a multicenter, qualitative Italian study
Source: Neurol Sci. 2023 Oct 10;45(3):1145–54. doi: 10.1007/s10072-023-07087-y (PMC10858142; doi:10.1007/s10072-023-07087-y)
Supplement: Supplementary file 5 — Supplementary file5 (PDF 203 KB) [file 10072_2023_7087_MOESM5_ESM.pdf]

Supplementary File 5. Table of booklet revisions

Domain	Revision	Based on
**Pictures**	Six pictures out of seven changed	Patients
	The approved one promoted to cover	Expert panel
**Layout** (readability and visual clarity)	Font changed from Calibri 13.5 to Helvetica 12	Patients, significant others, healthcare professionals
	Sections' titles and relevant headings put in magenta bold	
	More space allowed between paragraphs	
	All the explanation and example pages in a warm and light sand background, different from the white for introduction and boxes to complete the ACP document	Patients, significant others
	The box on previous completion of an Advance Directives document, moved to page 26 (before the ACP document); a clarification note added	Significant other
	All the sections of the legal ACP document placed on the even pages (except for the last one, page 30)	Patients, significant others
	The free text box "My ACP, my choices" (page 10, provisional version) removed	Patients, significant other
**Text**	The statement "MS is a chronic disease with a variable life span reduction between 7 and 14 years compared to the general population" replaced with "MS is a chronic disease with significant variability in its course and a variable life span reduction compared with the general population"	Patient
	Improved consistency in wording and expressions	Expert panel
	Repetitions avoided or sentences rephrased	Patients, healthcare professionals
	Replacement of the present tense with future tense in	Expert panel

	some sentences	
	The statement “To make your ACP document legally valid, you have to sign it” moved from page 4 of the provisional version to page 28	Expert panel
	The box “My values and ideas on life and future” moved from page 5 (provisional version) to page 30; heading changed into “Additional notes”	Patients, significant others, expert panel
**Response option**	The Likert scale improved by adding numbers, and the anchors put more straightforwardly (page 17)	Patients, health professionals
	Multiple choices allowed in sections describing preferences for the EOL period	Patients, significant other
	Page 23, section “Thinking about my end of life”, re-ordered and response options/boxes re-organised	Significant other, expert panel
